# Synthesis and crystal structure analysis of substituted bi­cyclo­[3.3.1]nona­nones

**DOI:** 10.1107/S2056989025003299

**Published:** 2025-04-17

**Authors:** Julien A. König, Bernd Morgenstern, Johann Jauch

**Affiliations:** a Organic Chemistry II, Saarland University, 66123 Saarbrücken, Germany; b Service Center X-ray diffraction, Saarland University, 66123 Saarbrücken, Germany; University of Missouri-Columbia, USA

**Keywords:** polycyclic, polyprenylated acyl­phloroglucinol, PPAPs, bridgehead substitution, carbocycles, crystal structure

## Abstract

A set of novel bi­cyclo­[3.3.1]nona­nones, namely, 4-methoxybi­cyclo­[3.3.1]non-3-ene-2,9-dione, C_10_H_12_O_3_ (**1**), 4,9,9-tri­methoxybi­cyclo­[3.3.1]non-3-en-2-ol, C_12_H_20_O_4_ (**2**), 4-meth­oxy-6-methyl-1-(3-methyl­but-2-en-1-yl)-6-(4-methyl­pent-3-en-1-yl)bi­cyclo­[3.3.1]non-3-ene-2,9-dione, C_22_H_32_O_3_ (**3**) and 4-(*tert*-but­yl)-4-hy­droxy-2-meth­oxy-8-methyl-7-(3-methyl­but-2-en-1-yl)-8-(4-methyl­pent-3-en-1-yl)bi­cyclo­[3.3.1]non-2-en-9-one, C_26_H_42_O_3_ (**4**), were synthesized and structurally elucidated by NMR, HRMS and X-ray crystallography.

## Abstract 2

A set of novel bi­cyclo­[3.3.1]nona­nones were synthesized and structurally elucidated by NMR, HRMS and X-ray crystallography.

## 1. Chemical context

Polycyclic polyprenylated acyl­phloroglucinols (PPAPs) are a class of structurally com­plex natural products predominantly isolated from plants of the *Hypericum* and *Garcinia* genera. Characterized by a highly oxygenated polycyclic core densely decorated with various substituents, these com­pounds exhibit remarkable chemical diversity and biological activity (Yang *et al.*, 2018 ▸). Notably, a single representative can already cover a wide range of different activities. The most important of these may include anti-inflammatory, anti­bacterial and anti­viral activity, as well as cytotoxicity, anti­tumour properties and use as an anti­depressant and neuroprotective agent. Hyperforin, the most prominent and best-studied PPAP to date, may serve as an example of the latter point in particular (Richard, 2014 ▸). The pronounced bicyclic framework, common to most PPAPs and many other natural com­pounds (Roy *et al.*, 2023 ▸), makes them particularly com­pelling for research in natural product chemistry and medicinal applications.
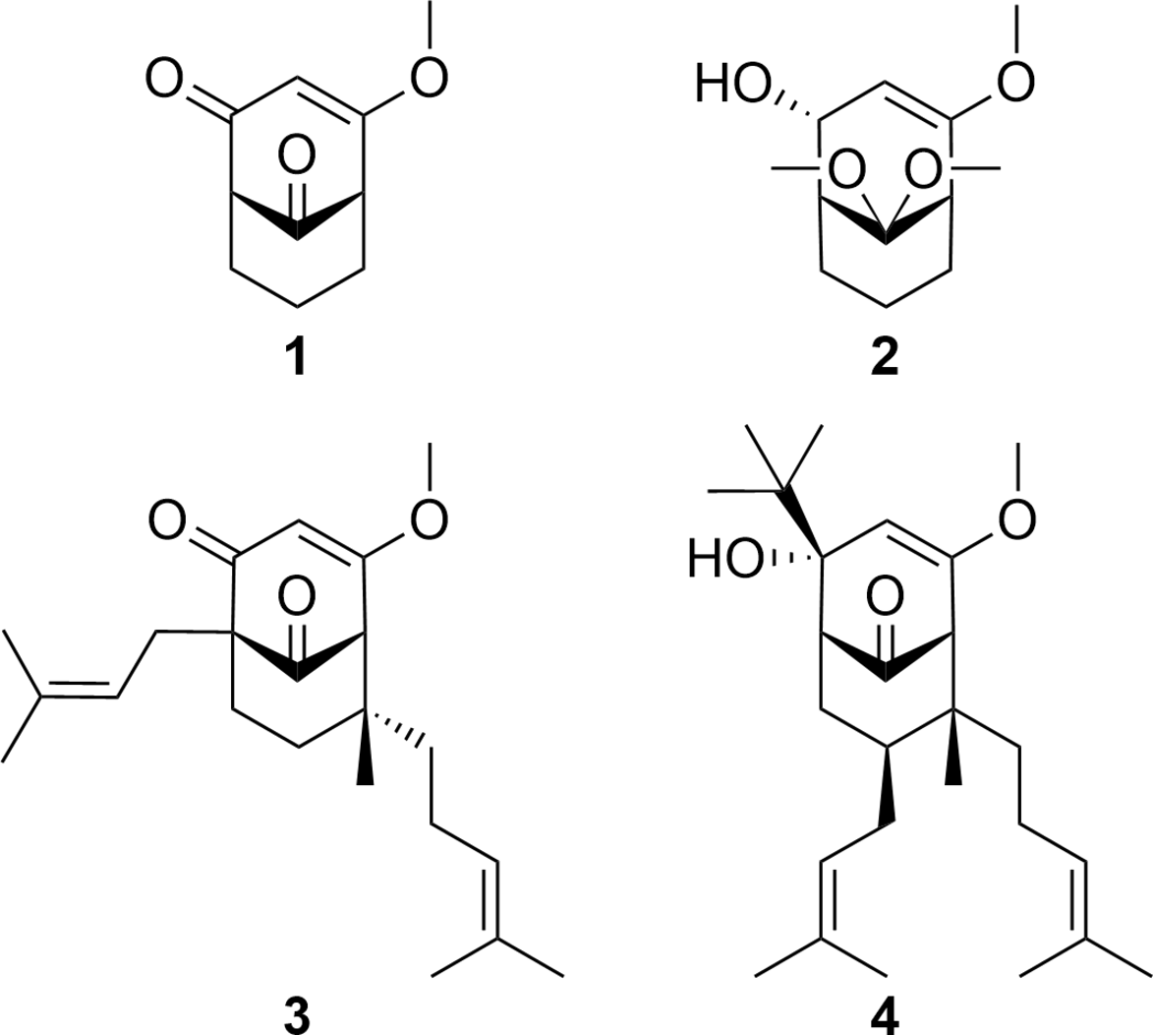


Several approaches have been explored to synthesize polycyclic polyprenylated acyl­phloroglucines (PPAPs), yet achieving regioselective and diastereoselective control during modification of the core structure can be challenging due to the mol­ecule’s dense stereochemically rich framework. Con­se­quently, precursors and inter­mediates often require rigorous confirmation of stereochemistry through advanced techniques, such as single-crystal X-ray diffraction, prior to further derivatization. Recent advancements in these stereochemical control strategies have opened new pathways not only to natural PPAPs but also to synthetic analogues with enhanced or modified bioactivity profiles.

In our approach to PPAP synthesis (König *et al.*, 2024 ▸, 2025 ▸), we focused on designing inter­mediate structures with a high degree of flexibility in substitution patterns, particularly at bridgehead positions. This flexibility is essential for achieving the precise stereochemical and functional com­plexity required for both natural and synthetic PPAPs, ultimately enhancing the efficiency and specificity of our synthetic route.

The title com­pounds **1**–**4** were synthesized as model structures to investigate the reductive and substitutional reactivity of the β-alk­oxy enone system found in PPAP precursors.

## 2. Structural commentary

**Figure 1:**
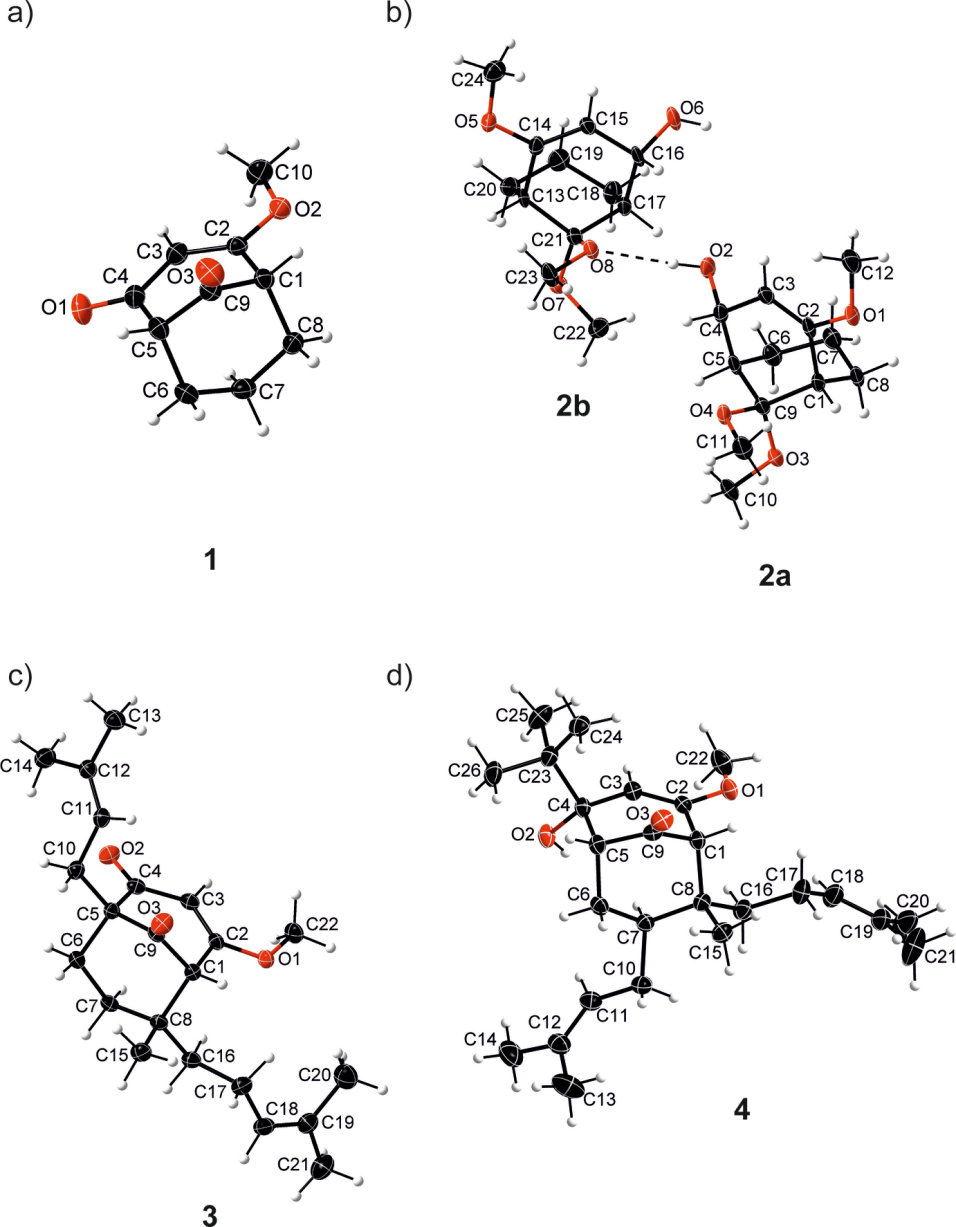
The mol­ecular structures of com­pounds (*a*) **1**, (*b*) **2a** and **2b**, (*c*) **3** and (*d*) **4**, with the atom labelling and displacement ellipsoids drawn at the 50% probability level. The dashed line in part (*b*) indicates the inter­molecular hy­dro­gen bond between the enanti­omeric com­pounds **2a** and **2 b**.

**Figure 2:**
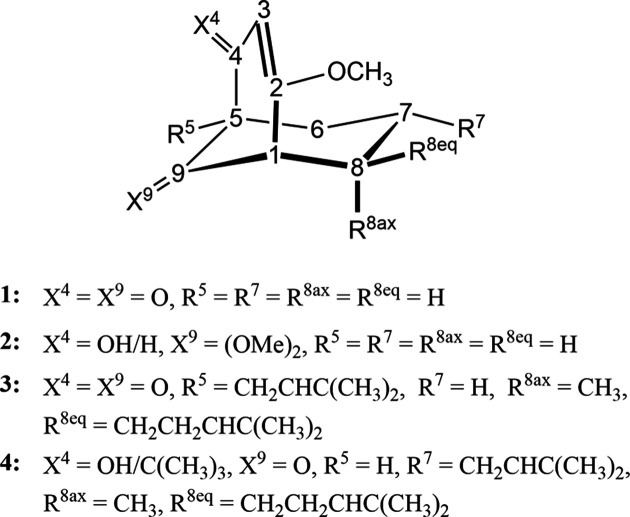
Numbering scheme for ring **I** (C1/C8/C7/C6/C5/C9) and ring **II** (C1–C5/C9) for the basic bi­cyclo­[3.3.1]nona­none framework and its substituents *X*^4^, *X*^9^, *R*^5^, *R*^7^, *R*^8ax^ and *R*^8eq^ for the assignment of the mol­ecular com­pounds **1**, **2**, **3** and **4**.

Crystals suitable for X-ray diffraction analysis were obtained for **1**–**4** and their mol­ecular structures are illustrated in Fig. 1 ▸. Compound **1** crystallizes in the Sohnke space group *P*2_1_ and was refined as an inversion twin. Compounds **2**, **3** and **4** crystallize in centrosymmetric space groups (*P*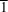, *P*2_1_/*n* and *P*2_1_/*c*, respectively) and thus occur as enanti­omeric pairs in the crystal. Notably, although com­pound **2** crystallizes in *P*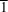, the asymmetric unit contains two crystallographically distinct mol­ecules (*Z*′ = 2), **2a** and **2b**, which are chemically mirror images of each other [Fig. 1 ▸(*b*)]. Both com­pounds are related to their respective crystallographically enanti­omeric partners through the inversion centre in the unit cell. The five mol­ecular com­pounds **1**, **2a**, **2b**, **3** and **4** share a bi­cyclo­[3.3.1]nona­none core structure but differ structurally at positions *X*^4^, *X*^9^, *R*^5^, *R*^7^, *R*^8eq^ and *R*^8ax^ (Fig. 2 ▸). In the crystal structures of com­pounds **2** and **4**, the OH groups form a hy­dro­gen-bonded network.

**Figure 3:**
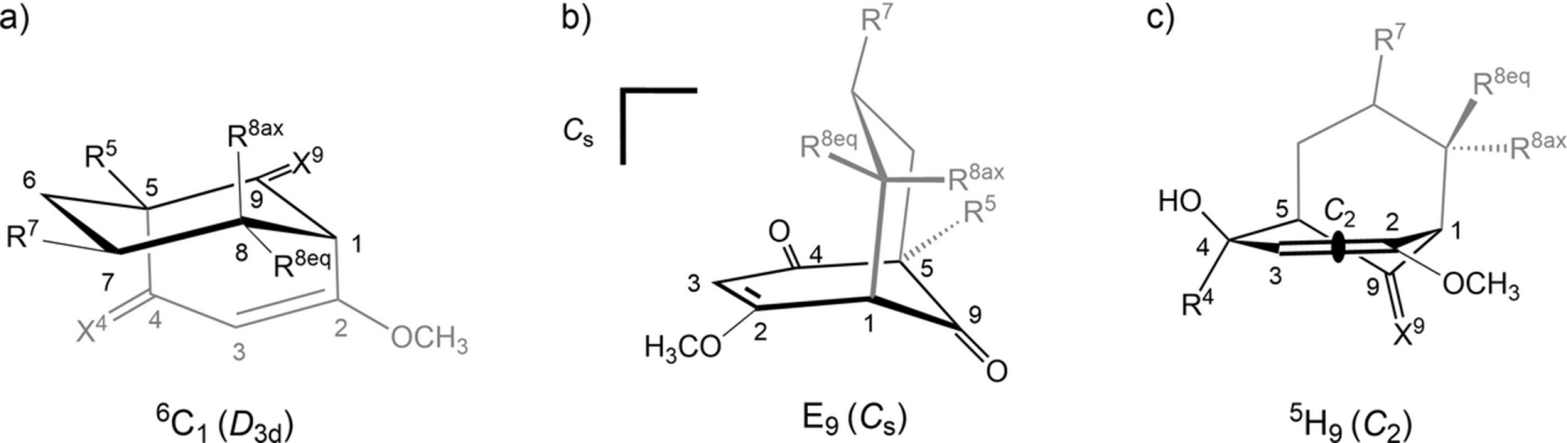
Overview of the cyclo­hexane conformations (bold black bonds) and their symmetries as described in the text: (*a*) chair conformation (*D*_3*d*_), with four atoms in the plane and atom C6 above and C1 below (^6^C_1_); (*b*) envelope conformation (*C*_s_) with atoms C1–C5 in the plane and C9 below (E_9_); (*c*) half-chair conformation (*C*_2_) with atoms C1–C4 in the plane and atom C5 above and C9 below (looking towards the twofold axis in the middle of bond C2—C3 and bond C5—C9).

To describe the structural characteristics of these mol­ecules, puckering parameters (Cremer & Pople, 1975 ▸) were analyzed (Table 1 ▸). For consistent representation, all crystal structures were treated with a unified naming scheme for the bi­cyclo­[3.3.1]nonane core (Fig. 2 ▸). The starting atom for rings **I** and **II** is C1, with the rotation direction chosen as C1 toward C8 for ring **I** and C1 toward C2 for ring **II**. Focus was placed on the folding of the two six-membered rings C1/C8/C7/C6/C5/C9 (**I**) and C1–C5/C9 (**II**). The influence of substituents of ring **I** on its expected chair conformation [^6^C_1_; Fig. 3 ▸(*a*)] was determined using the puckering parameters. With regard to ring **II**, it is known that the introduction of three *sp*^2^-hybridized atoms into the bi­cyclo­[3.3.1]nonane skeleton (*X*^4^ = O) leads to planarity of this part of the ring (Zefirov & Palyulin, 1991 ▸). Consequently one would expect a *C*_s_-symmetric envelope conformation (E_9_) for the six-membered ring in which there are five C atoms in the plane and C9 below [Fig. 3 ▸(*b*)]. However, replacing the *sp*^2^-hybridized C atom at position 4 of ring **II** with an *sp*^3^-hybridized C atom (*X*^4^ = OH/H) alters the folding of the respective ring, leading to a *C*_2_-symmetric half-chair conformation (^5^H_9_). In this conformation, four C atoms lie in the plane, with C5 positioned above and C9 below it [Fig. 3 ▸(*c*)].

The puckering parameters *Q*, Θ and Φ allow a com­plete description of the conformations of **I** and **II** in polar coordinates, with every possible conformation represented as a point on a sphere of radius *Q*, the polar angle Θ [angle with respect to the positive polar axis (the north pole) with 0 ≤ Θ ≤ 180°], and the azimuthal angle Φ (rotation around the equator with 0 ≤ Φ ≤ 360°) (Cremer & Pople, 1975 ▸; Giacovazzo *et al.*, 2011 ▸). On this sphere, the ideal chair conformation (C) (*D*_3*d*_) is located at the poles with Θ = 0° and Θ = 180° (Φ undefined). The two higher-energy states, half-chair (H) (*C*_2_) and envelope (E) (*C*_s_), are situated towards the equator at tan Θ = ±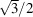 (Θ = 50.8°) and tan Θ = ±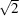 (Θ = 54.7°), and inter­convert *via* pseudorotation [Φ = *n* × 60° + 30° for (H) and Φ = *n* × 60° for (E)]. Table 1 ▸ summarizes the puckering parameters for rings **I** and **II**. In the five investigated structures **1**, **2a**, **2b**, **3** and **4** of the bi­cyclo­[3.3.1]nonane core, all conformations of the six-membered rings **I** are very close to the ideal chair conformation (^6^C_1_), with very tightly grouped Θ values (169.6 < Θ < 172.2°). In accordance with a previous report (Zefirov & Palyulin, 1991 ▸), the influence of substituents *R*^5^, *R*^7^, *R*^8eq^ and *R*^8ax^, as well as the difference between the keto group at C9 in com­pounds **1**, **3** and **4** com­pared to the dimeth­oxy group in **2**, appears to have no significant impact on the ^6^C_1_ conformation of ring **I**. Even bulky substituents in the axial position at C7, for example, OCH_2_Ph [Inouye *et al.*, 1987 ▸; *Q* = 0.561 (3) Å, Θ = 165.0 (3) and Φ = 118.1 (13)°] or CH_2_CHC(CH_3_)_2_ [Biber *et al.*, 2011 ▸; *Q* = 0.560 Å, Θ = 170.8 (5) and Φ = 128 (3)°] leave the six-membered ring **I** in a nearly ideal chair conformation with a small distortion towards ^7^E.

A closer examination of the puckering parameters for ring **II** reveals a slightly different picture. Compounds **1** and **3** with *X*^4^ = O adopt a nearly ideal envelope conformation (E_9_) (**1**: Θ = 56.7° and Φ = 306.0°; **3**: Θ = 58.2° and Φ = 310.9°), with C9 lying out of the plane of the ring and a mirror plane passing through atoms C3 and C9 [Fig. 3 ▸(*b*)]. When the C4 keto group is reduced to an alcohol (*sp*^2^→*sp*^3^), the ring exhibits greater flexibility. In this case, com­pound **2** adopts a conformation close to the half-chair form (^5^H_9_) (**2a**: Θ = 48.3° and Φ = 284.1°; **2b**: Θ = 47.8° and Φ = 286.9°), with a twofold axis passing through the bonds C2—C3 and C5—C9 [Fig. 3 ▸(*c*)]. In contrast, com­pound **4** is better described as a linear combination of E_9_ and ^5^H_9_ (Θ = 52.4° and Φ = 298.5°).

## 3. Supra­molecular features

**Figure 4:**
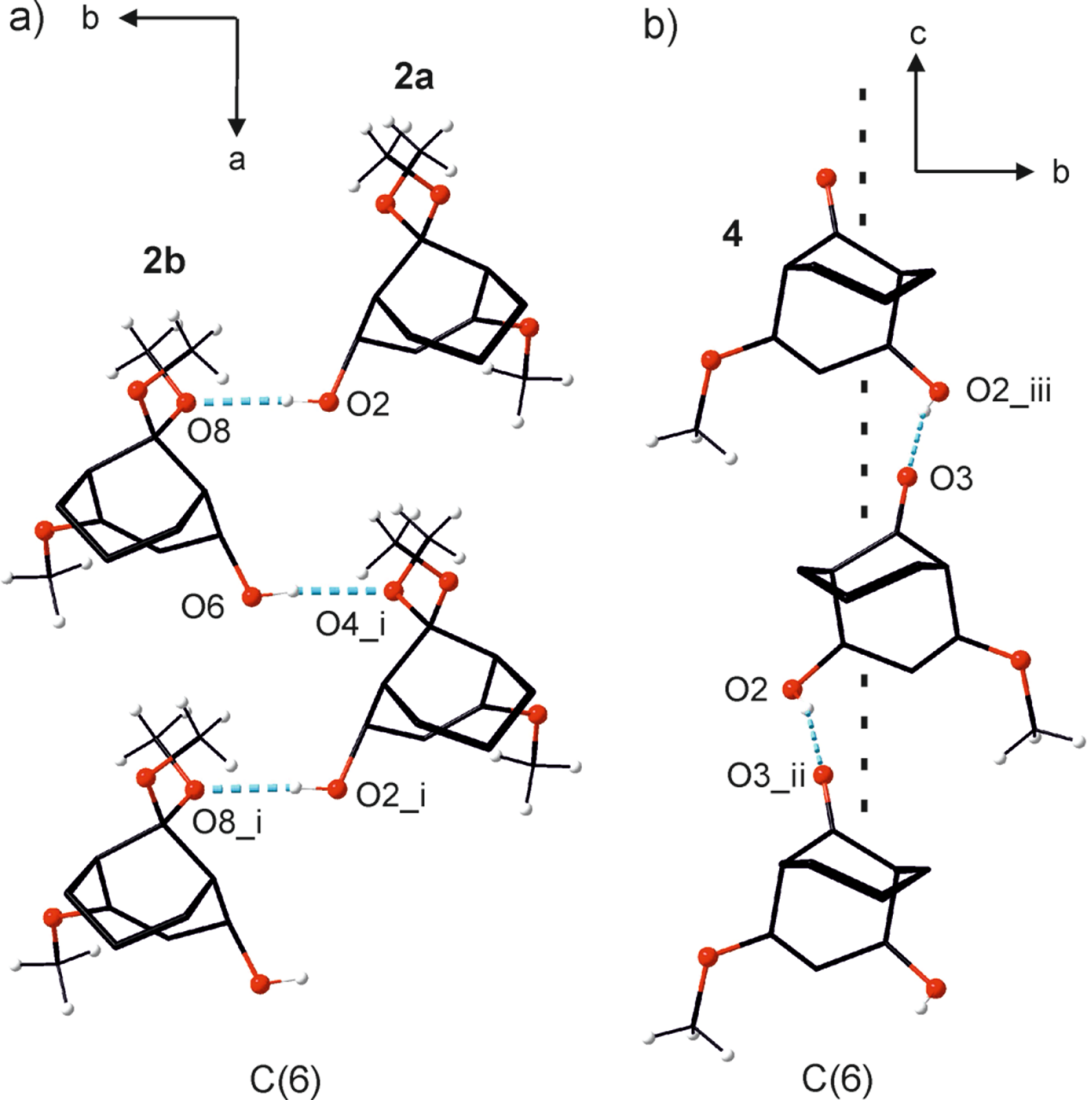
Hydrogen-bonding network of **2** and **4**. (*a*) Hydrogen bonds (blue dashed lines) perpendicular to the *a* direction between **2a** and **2b***via* O2—H20 and O8, and O6—H6O and O4^i^ [symmetry code: (i) *x* + 1, *y*, *z*]. The formed *C*(6) chain is aligned in *a* direction. (*b*) The hy­dro­gen bonds (blue dashed lines) in the *c* direction *via* O2—H2 and O3^ii^ [symmetry code: (ii) *x*, −*y* + 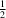, *z* − 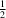 and *via* O2^iii^—H2^iii^ and O3 [symmetry code: (iii) *x*, −*y* + 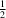, *z* + 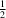], are deflected by 12.40 (4)° against the *c*-glide plane (black dashed line). The formed *C*(6) chain has the symmetry of the Frieze group *p*11g.

Compounds **2** and **4** feature an OH group at C4, with the H atom capable of acting as a donor in C—H⋯O hy­dro­gen bonding (Tables 2 ▸ and 3 ▸). In the crystal structure of **2**, there are two crystallographically independent mol­ecules in the asymmetric unit, which are chemically enanti­omers of each other. **2a** and **2b** are inter­connected *via* hy­dro­gen bonds [Fig. 4 ▸(*a*)]. **2a** forms a hy­dro­gen bond perpendicular to the *a* axis and directed along the *b* axis, with H2O as the donor and O8 in **2b** as the acceptor [O2—H2O⋯O8 = 2.858 (3) Å and 176 (3)°]. Respectively, **2b** forms another hy­dro­gen bond nearly parallel to the first (angle between lines O2⋯O8 and O6⋯O4^i^; symmetry code: (i) *x* + 1, *y*, *z*: 13.67 (8)°], with H6O as the donor and O4 in **2a** as the acceptor [O6—H6O⋯O4^i^; 2.863 (3)Å and 171 (3)°]. The distances between the strands are approximately equidistant [H6O⋯H2O = 3.82 (4) Å and H2O^i^⋯H6O = 3.92 (4) Å]. In both **2a** and **2b**, six atoms are involved in the bonding resulting in an infinite *C*(6) chain of hy­dro­gen-bonded mol­ecules along the *a* direction (Bernstein *et al.*, 1995 ▸). The crystal structure of **4** possesses a crystallographic glide mirror plane, which transforms the hy­dro­gen bonds between **4** with H2 as donor and its enanti­omer with O3^ii^ as acceptor [symmetry code: (ii) *x*, −*y* + 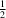, *z* − 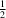] into one another [Fig. 4 ▸(*b*)]. In this case, the hy­dro­gen bonds are slightly tilted from the glide-plane in the *c* direction [angle between the line O3^ii^⋯O2 and the *c*-glide plane = 12.40 (4)°]. Here too an infinite *C*(6) chain forms. The symmetry class of this Frieze group is *p*11g (No. 5 in Inter­national Tables for Crystallography, 2010 ▸).

## 4. Database survey

A search of the Cambridge Structural Database (CSD, Version 5.45, November 2024; Groom *et al.*, 2016 ▸) indicated 441, 165 and 15 com­pounds incorporating a bi­cyclo­[3.3.1]non-2-ene, bi­cyclo­[3.3.1]non-2-en-9-one and 4-methoxybi­cyclo­[3.3.1]non-3-ene-2,9-dione motif, respectively.

## 5. Synthesis and crystallization

### 5.1. 4-Methoxybi­cyclo­[3.3.1]non-3-ene-2,9-dione (1)

The reaction was carried out in a round-bottomed flask under ambient conditions. To a solution of 4-hy­droxybi­cyclo­[3.3.1]non-3-ene-2,9-dione (97.9 mg, 0.59 mmol; Shishido *et al.*, 1986 ▸; Schönwälder *et al.*, 1984 ▸) in 6 ml of acetone was added K_2_CO_3_ (333 mg, 2.41 mmol) and dimethyl sulfate (70 µl, 0.74 mmol). The suspension was refluxed for 2 h. The reaction mixture was allowed to reach room tem­per­a­ture and treated with H_2_O. The layers were separated and the aqueous layer was extracted thrice with Et_2_O. The combined organic extracts were dried over anhydrous MgSO_4_ and concentrated *in vacuo*. Purification of the residue by flash column chromatography (^*n*^Pen/Et_2_O = 1:2) afforded **1** (yield: 84.2 mg, 0.47 mmol, 79%; m.p. 365.1–366.4 K) as a colourless solid.

^1^H NMR (CDCl_3_, 400 MHz): δ 5.79 (*s*, 1H), 3.79 (*s*, 3H), 3.21–3.19 (*m*, 2H), 2.22–2.16 (*m*, 1H), 2.14–2.07 (*m*, 1H), 2.00–1.86 (*m*, 2H), 1.79–1.60 (*m*, 2H); ^13^C NMR (CDCl_3_, 100 MHz): δ 207.4, 195.6, 175.6, 105.9, 61.3, 56.9, 53.3, 32.6, 30.5, 17.5.

HRMS (ESI) *m*/*z* calculated for C_10_H_12_O_3_^+^ [*M* + H]^+^: 181.08592, found: 181.08547.

### 5.2. 4,9,9-Tri­methoxybi­cyclo­[3.3.1]non-3-en-2-ol (2)

This com­pound was synthesized over two steps.

The reaction was carried out in a flame-dried round-bottomed flask under inert conditions. To a solution of 4-hydroxybi­cyclo­[3.3.1]non-3-ene-2,9-dione (504 mg, 3.02 mmol; Shishido *et al.*, 1986 ▸; Schönwälder *et al.*, 1984 ▸) in 30 ml of dry methanol was added PTSA (117 mg, 0.62 mmol). The reaction mixture was refluxed overnight. The reaction mixture was allowed to reach room tem­per­a­ture and concentrated *in vacuo*. Purification of the residue by flash column chromatography (^*n*^Pen/Et_2_O = 1:1) afforded 4,9,9-tri­methoxybi­cyclo­[3.3.1]non-3-en-2-one (yield: 593 mg, 2.62 mmol, 87%) as a colourless oil that solidifies in the cold.

^1^H NMR (CDCl_3_, 500 MHz): δ 5.47 (*s*, 1H), 3.64 (*s*, 3H), 3.12 (*s*, 3H), 3.05 (*s*, 3H), 2.74 (*q*, *J* = 3.2 Hz, 1H), 2.68–2.66 (*m*, 1H), 1.76 (*tdd*, *J* = 13.3, 5.1, 3.9 Hz, 1H), 1.66 (*tdd*, *J* = 13.6, 5.4, 4.4 Hz, 1H), 1.55 (*dddt*, *J* = 15.0, 4.7, 3.1, 1.6 Hz, 1H), 1.49 (*dddt*, *J* = 13.5, 5.0, 3.2, 1.6 Hz, 1H), 1.42–1.24 (*m*, 2H); ^13^C NMR (CDCl_3_, 125 MHz): δ 199.7, 176.6, 103.7, 100.7, 56.1, 48.8, 47.7, 46.8, 42.1, 24.1, 22.7, 16.4.

HRMS (ESI) *m*/*z* calculated for C_12_H_19_O_4_^+^ [*M* + H]^+^: 227.12779, found: 227.12863.

The reaction was carried out in a flame-dried round-bottomed flask under inert conditions. To a solution of 4,9,9-tri­methoxybi­cyclo­[3.3.1]non-3-en-2-one (704 mg, 3.11 mmol) in 60 ml of dry THF was added dropwise DIBAL-H (6.2 ml, 6.20 mmol, 1.0 *M* in hexa­ne) at 195 K. After stirring for 2 h, the reaction mixture was warmed to 233 K and then to 273 K. The reaction mixture was treated with an aqueous solution of potassium sodium tartrate. The layers were separated, and the aqueous layer was extracted thrice with Et_2_O. The combined organic extracts were dried over anhydrous MgSO_4_ and concentrated *in vacuo*. Purification of the residue by flash column chromatography (^*n*^Pen/Et_2_O = 1:1) afforded **2** (yield: 485 mg, 2.12 mmol, 69%; m.p. 336.8–337.4 K) as a colourless solid.

^1^H NMR (CDCl_3_, 400 MHz): δ 4.81 (*d*, *J* = 2.8 Hz, 1H), 4.57–4.52 (*m*, 1H), 3.54 (*s*, 3H), 3.19 (*s*, 3H), 3.16 (*s*, 3H), 2.52 (*q*, *J* = 3.0 Hz, 1H), 2.34–2.30 (*m*, 1H), 1.92–1.85 (*m*, 1H), 1.73 (*ttd*, *J* = 12.6, 4.4, 1.1 Hz, 1H), 1.61–1.31 (*m*, 5H); ^13^C NMR (CDCl_3_, 100 MHz): δ 155.5, 101.7, 98.7, 68.6, 54.7, 47.6, 46.8, 40.5, 38.4, 24.7, 21.8, 15.75.

HRMS (ESI) *m*/*z* calculated for C_12_H_19_O_4_^−^ [*M* – H]^−^: 227.12888,found: 227.12864.

### 5.3. 4-Meth­oxy-6-methyl-1-(3-methyl­but-2-en-1-yl)-6-(4-methyl­pent-3-en-1-yl)bi­cyclo­[3.3.1]non-3-ene-2,9-dione (3)

The reaction was carried out in a flame-dried round-bottomed flask under inert conditions. To a solution of 4-meth­oxy-6-methyl-6-(4-methyl­pent-3-en-1-yl)bi­cyclo­[3.3.1]non-3-ene-2,9-dione (55.4 mg, 0.20 mmol; König *et al.*, 2024 ▸) and prenyl bromide (1.1 ml, 9.52 mmol) in 2.2 ml dry THF was added over 3 min a freshly prepared solution of LDA (1.6 ml, 0.40 mmol) at 173 K. The now yellow solution was allowed to reach 195 K over 30 min and then treated with H_2_O. The layers were separated, and the aqueous layer was extracted thrice with Et_2_O. The combined organic extracts were dried over anhydrous MgSO_4_ and concentrated *in vacuo*. Purification of the residue by flash column chromatography (^*n*^Pen/Et_2_O = 3:1→1:1) afforded **3** (yield: 9.6 mg, 27.9 µmol, 14%; m.p. 341.9–342.2 K) as a colourless solid.

^1^H NMR (CDCl_3_, 400 MHz): δ 5.71 (*s*, 1H), 5.01 (tsept, *J* = 7.0, 1.2 Hz, 1H), 4.97 (tsept, *J* = 7.0, 1.2 Hz, 1H), 3.74 (*s*, 3H), 2.99 (*s*, 1H), 2.42 (*d*, *J* = 6.8 Hz, 2H), 2.08 (*tt*, *J* = 12.5, 6.1 Hz, 1H), 1.90 (*tt*, *J* = 12.7, 6.6 Hz, 1H), 1.82–1.78 (*m*, 2H), 1.76–1.69 (*m*, 1H), 1.68 (*s*, 3H), 1.66 (*s*, 3H), 1.63 (*s*, 3H), 1.60 (*s*, 3H), 1.38–1.32 (*m*, 1H), 1.22 (*td*, *J* = 11.3, 5.1 Hz, 2H), 1.00 (*s*, 3H); ^13^C NMR (CDCl_3_, 100 MHz): δ 207.9, 197.8, 174.9, 133.8, 131.6, 124.0, 119.6, 106.0, 63.1, 62.6, 56.4, 42.4, 41.7, 34.5, 31.2, 29.3, 25.9, 25.7, 21.9, 21.3, 18.0, 17.5.

HRMS (ESI) *m*/*z* calculated for C_22_H_33_O_3_^+^ [*M* + H]^+^: 345.24242, found: 345.24241.

### 5.4. 4-*tert*-Butyl-4-hy­droxy-2-meth­oxy-8-methyl-7-(3-methyl­but-2-en-1-yl)-8-(4-methyl­pent-3-en-1-yl)bi­cyclo­[3.3.1]non-2-en-9-one (4)

The reaction was carried out in a flame-dried round-bottomed flask under inert conditions. To a solution of 4-meth­oxy-6-methyl-7-(3-methyl­but-2-en-1-yl)-6-(4-methyl­pent-3-en-1-yl)bi­cyclo­[3.3.1]-non-3-ene-2,9-dione (17.4 mg, 50.5 µmol; König *et al.*, 2025 ▸) in 5 ml of dry THF was added dropwise *t*-BuLi (53 µl, 101 µmol) at 168 K. The reaction mixture was stirred for 90 min and then treated with a saturated aqueous solution of NH_4_Cl. The layers were separated, and the aqueous layer was extracted thrice with Et_2_O. The combined organic extracts were dried over anhydrous MgSO_4_ and concentrated *in vacuo*. Purification of the residue by flash column chromatography (^*n*^Pen/Et_2_O = 10:1) afforded **4** (yield: 19.0 mg, 47.3 µmol, 94%; m.p. 382.7.1–384.3 K) as a colourless solid.

^1^H NMR (CDCl_3_, 400 MHz): δ 5.07–5.01 (*m*, 3H), 3.56 (*s*, 3H), 2.83 (*bs*, 1H), 2.70 (*s*, 1H), 2.29 (*ddd*, *J* = 14.3, 3.9 Hz, 2.7 Hz, 1H), 2.23–2.16 (*m*, 1H), 2.10 (*dd*, *J* = 13.7, 4.9 Hz, 1H), 1.85–1.77 (*m*, 1H), 1.68 (*s*, 6H), 1.65–1.62 (*m*, 1H), 1.61 (*s*, 3H), 1.57 (*s*, 3H), 1.54–1.39 (*m*, 3H), 1.22–1.15 (*m*, 1H), 0.92 (*s*, 9H), 0.84 (*s*, 3H); ^13^C NMR (CDCl_3_, 100 MHz): δ 212.0, 155.4, 132.7, 131.0, 124.9, 123.1, 101.6, 77.5, 58.6, 54.6, 52.2, 46.2, 41.2, 39.8, 38.5, 32.5, 28.2, 25.8, 25.7, 25.3, 21.7, 17.9, 17.5, 17.3.

HRMS (ESI) *m*/*z* calculated for C_26_H_41_O_3_^−^ [*M* – H]^−^: 401.30612, found: 401.30503.

All com­pounds were crystallized after flash chromatography by dissolving 10 mg to 30 mg of the respective purified com­pound in a 10 ml round-bottomed flask with 1–2 ml DCM. The flasks were topped with a septum and a cannula to allow slow solvent evaporation. All solutions were left undisturbed at room tem­per­a­ture for two to four weeks to yield colourless to pale yellow crystals.

## 6. Refinement

Crystal data, data collection and structure refinement details are summarized in Table 4 ▸. All H atoms were treated as recommended by Müller *et al.* (2006 ▸). A riding model was used for the C-bonded H atoms, with *U*_iso_(H) = 1.5*U*_eq_(meth­yl C) and 1.2*U*_eq_(C) for other C-bound H atoms. The positional parameters of the O-bonded H atoms of **2a**, **2b** and **4** were refined using isotropic displacement parameters which were set at 1.5 times the *U*_eq_ value of the parent atom. In addition, restraints of 0.84 (1) Å were used for the O—H bond lengths. The crystal structure of com­pound **1** was refined as an inversion twin (BASF = 0.5).

## Supplementary Material

Crystal structure: contains datablock(s) 1, 2, 3, 4, global. DOI: 10.1107/S2056989025003299/ev2016sup1.cif

Supporting information file. DOI: 10.1107/S2056989025003299/ev20161sup2.cml

Supporting information file. DOI: 10.1107/S2056989025003299/ev20162sup3.cml

Supporting information file. DOI: 10.1107/S2056989025003299/ev20163sup4.cml

Supporting information file. DOI: 10.1107/S2056989025003299/ev20164sup5.cml

Structure factors: contains datablock(s) 1. DOI: 10.1107/S2056989025003299/ev20161sup6.hkl

Structure factors: contains datablock(s) 2. DOI: 10.1107/S2056989025003299/ev20162sup7.hkl

Structure factors: contains datablock(s) 3. DOI: 10.1107/S2056989025003299/ev20163sup8.hkl

Structure factors: contains datablock(s) 4. DOI: 10.1107/S2056989025003299/ev20164sup9.hkl

CCDC references: 2429999, 2430008, 2430001, 2430007

Additional supporting information:  crystallographic information; 3D view; checkCIF report

## Figures and Tables

**Table 1:** Puckering parameters Q (Å), Θ (°) and Φ (°) of the cyclo­hexane rings **I** and **II** of **1**, **2a**, **2b**, **3** and **4**

		Ring **I**			Ring **II**	
	*Q* (Å)	Θ (°)	Φ (°)	*Q* (Å)	Θ (°)	Φ (°)
**1**	0.590 (2)	172.2 (2)	104.4 (1)	0.505 (2)	56.7 (2)	306.0 (2)
**2a**	0.591 (3)	169.6 (3)	140.4 (2)	0.534 (3)	48.3 (3)	284.1 (4)
**2b** ^*a*^	0.593 (3)	170.7 (3)	139.1 (2)	0.541 (3)	47.8 (3)	286.9 (4)
**3**	0.592 (2)	171.6 (2)	115.6 (1)	0.501 (2)	58.2 (2)	310.9 (2)
**4**	0.592 (1)	170.2 (1)	120.6 (7)	0.489 (1)	52.4 (1)	298.5 (2)

Note: (*a*) for **2b** (the enanti­omer of **2a**), the tabulated values of Θ and Φ were calculated from the observed Θ′ and Φ′ using the equations Θ = 180° – Θ′ and Φ = 180° + Φ′.

**Table 2:** Hydrogen-bond geometry (Å, °) for **2**[Chem scheme1]

*D*—H⋯*A*	*D*—H	H⋯*A*	*D*⋯*A*	*D*—H⋯*A*
O6—H6O⋯O4^i^	0.84 (1)	2.03 (1)	2.863 (3)	172 (3)
O2—H2O⋯O8	0.84 (1)	2.02 (1)	2.858 (3)	176 (3)

Symmetry code: (i) 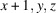.

**Table 3:** Hydrogen-bond geometry (Å, °) for **4**[Chem scheme1]

*D*—H⋯*A*	*D*—H	H⋯*A*	*D*⋯*A*	*D*—H⋯*A*
O2—H2⋯O3^i^	0.85 (1)	2.06 (1)	2.8736 (11)	162 (2)

Symmetry code: (i) 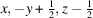.

**Table 4:** Experimental details Experiments were carried out with Mo *K*α radiation using a Bruker D8 VENTURE PHOTON II. Absorption was corrected for by multi-scan methods (*SADABS*; Krause *et al.*, 2015 ▸).

	(1)	(2)	(3)	(4)
Crystal data				
Chemical formula	C_10_H_12_O_3_	C_12_H_20_O_4_	C_22_H_32_O_3_	C_26_H_42_O_3_
*M*_r_	180.20	228.28	344.47	402.59
Crystal system, space group	Monoclinic, *P*2_1_	Triclinic, *P* 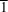	Monoclinic, *P*2_1_/*n*	Monoclinic, *P*2_1_/*c*
Temperature (K)	153	152	133	143
*a*, *b*, *c* (Å)	6.4819 (4), 7.4766 (5), 9.0627 (5)	7.7330 (15), 10.976 (2), 13.874 (3)	14.1871 (6), 6.3306 (2), 22.4159 (8)	15.1165 (5), 13.9068 (4), 12.8150 (4)
α, β, γ (°)	90, 99.546 (2), 90	101.984 (5), 92.032 (5), 90.293 (5)	90, 101.702 (1), 90	90, 113.218 (1), 90
*V* (Å^3^)	433.12 (5)	1151.2 (4)	1971.39 (13)	2475.81 (13)
*Z*	2	4	4	4
μ (mm^−1^)	0.10	0.10	0.08	0.07
Crystal size (mm)	0.38 × 0.08 × 0.06	0.28 × 0.24 × 0.04	0.18 × 0.14 × 0.04	0.26 × 0.16 × 0.08

Data collection				
*T*_min_, *T*_max_	0.699, 0.746	0.578, 0.746	0.687, 0.746	0.704, 0.746
No. of measured, independent and observed [*I* > 2σ(*I*)] reflections	8258, 2031, 1948	8885, 4662, 2677	28995, 4348, 3271	87917, 5484, 4697
*R*_int_	0.028	0.055	0.067	0.048
(sin θ/λ)_max_ (Å^−1^)	0.658	0.625	0.642	0.642

Refinement				
*R*[*F*^2^ > 2σ(*F*^2^)], *wR*(*F*^2^), *S*	0.030, 0.074, 1.08	0.057, 0.139, 0.98	0.046, 0.116, 1.05	0.040, 0.108, 1.05
No. of reflections	2031	4662	4348	5484
No. of parameters	119	301	232	274
No. of restraints	1	2	0	1
H-atom treatment	H-atom parameters constrained	H atoms treated by a mixture of independent and constrained refinement	H-atom parameters constrained	H atoms treated by a mixture of independent and constrained refinement
Δρ_max_, Δρ_min_ (e Å^−3^)	0.18, −0.15	0.24, −0.24	0.28, −0.22	0.29, −0.20
Absolute structure	Refined as an inversion twin	–	–	–
Absolute structure parameter	0.5	–	–	–

Computer programs: *APEX4* and *SAINT* (Bruker, 2021 ▸), *SHELXT2018* (Sheldrick, 2015*a* ▸), *SHELXL2019* (Sheldrick, 2015*b* ▸), *ShelXle* (Hübschle *et al.*, 2011 ▸), *DIAMOND* (Brandenburg, 2020 ▸) and *publCIF* (Westrip, 2010 ▸).

## supplementary crystallographic information

### 4-Methoxybicyclo[3.3.1]non-3-ene-2,9-dione (1) Crystal data

**Table:**

C_10_H_12_O_3_	*F*(000) = 192
*M**_r_* = 180.20	*D*_x_ = 1.382 Mg m^−^^3^
Monoclinic, *P*2_1_	Mo *K*α radiation, λ = 0.71073 Å
*a* = 6.4819 (4) Å	Cell parameters from 6064 reflections
*b* = 7.4766 (5) Å	θ = 2.3–27.9°
*c* = 9.0627 (5) Å	µ = 0.10 mm^−^^1^
β = 99.546 (2)°	*T* = 153 K
*V* = 433.12 (5) Å^3^	Rod, colourless
*Z* = 2	0.38 × 0.08 × 0.06 mm

### 4-Methoxybicyclo[3.3.1]non-3-ene-2,9-dione (1) Data collection

**Table:**

Bruker D8 VENTURE PHOTON II diffractometer	1948 reflections with *I* > 2σ(*I*)
Radiation source: INCOATEC IÂµS microfocus sealed tube	*R*_int_ = 0.028
φ and ω scans	θ_max_ = 27.9°, θ_min_ = 2.3°
Absorption correction: multi-scan (SADABS; Krause *et al.*, 2015)	*h* = −8→7
*T*_min_ = 0.699, *T*_max_ = 0.746	*k* = −9→9
8258 measured reflections	*l* = −11→11
2031 independent reflections	

### 4-Methoxybicyclo[3.3.1]non-3-ene-2,9-dione (1) Refinement

**Table:**

Refinement on *F*^2^	Secondary atom site location: difference Fourier map
Least-squares matrix: full	Hydrogen site location: inferred from neighbouring sites
*R*[*F*^2^ > 2σ(*F*^2^)] = 0.030	H-atom parameters constrained
*wR*(*F*^2^) = 0.074	*w* = 1/[σ^2^(*F*_o_^2^) + (0.0364*P*)^2^ + 0.0624*P*] where *P* = (*F*_o_^2^ + 2*F*_c_^2^)/3
*S* = 1.08	(Δ/σ)_max_ < 0.001
2031 reflections	Δρ_max_ = 0.18 e Å^−^^3^
119 parameters	Δρ_min_ = −0.15 e Å^−^^3^
1 restraint	Absolute structure: Refined as an inversion twin
Primary atom site location: structure-invariant direct methods	Absolute structure parameter: 0.5

### 4-Methoxybicyclo[3.3.1]non-3-ene-2,9-dione (1) Special details

**Table:**

Geometry. All esds (except the esd in the dihedral angle between two l.s. planes) are estimated using the full covariance matrix. The cell esds are taken into account individually in the estimation of esds in distances, angles and torsion angles; correlations between esds in cell parameters are only used when they are defined by crystal symmetry. An approximate (isotropic) treatment of cell esds is used for estimating esds involving l.s. planes.
Refinement. Refined as a 2-component inversion twin

### 4-Methoxybicyclo[3.3.1]non-3-ene-2,9-dione (1) Fractional atomic coordinates and isotropic or equivalent isotropic displacement parameters (Å^2^)

**Table:**

	*x*	*y*	*z*	*U*_iso_*/*U*_eq_	
O1	0.6356 (2)	0.20219 (18)	0.73144 (16)	0.0358 (3)	
O2	0.80123 (19)	0.73388 (16)	0.49209 (12)	0.0255 (3)	
O3	0.2740 (2)	0.7098 (2)	0.73484 (15)	0.0345 (3)	
C1	0.6305 (3)	0.7548 (2)	0.70026 (17)	0.0214 (3)	
H1	0.578127	0.868685	0.649526	0.026*	
C2	0.7203 (2)	0.6369 (2)	0.59258 (16)	0.0207 (3)	
C10	0.8884 (3)	0.6368 (3)	0.3793 (2)	0.0318 (4)	
H10A	0.930016	0.721305	0.306964	0.048*	
H10B	0.783321	0.554093	0.327603	0.048*	
H10C	1.011125	0.569139	0.426813	0.048*	
C3	0.7170 (3)	0.4564 (2)	0.59890 (18)	0.0241 (3)	
H3	0.773987	0.388629	0.526623	0.029*	
C4	0.6278 (3)	0.3647 (2)	0.71463 (18)	0.0242 (3)	
C5	0.5211 (3)	0.4780 (2)	0.82067 (18)	0.0240 (3)	
H5	0.397653	0.412087	0.846504	0.029*	
C6	0.6779 (3)	0.5196 (2)	0.96573 (19)	0.0285 (4)	
H6A	0.604837	0.586413	1.036072	0.034*	
H6B	0.729428	0.405919	1.014438	0.034*	
C7	0.8636 (3)	0.6296 (2)	0.93294 (18)	0.0277 (4)	
H7A	0.951032	0.665124	1.028598	0.033*	
H7B	0.950543	0.554829	0.877127	0.033*	
C8	0.7938 (3)	0.7970 (2)	0.84179 (19)	0.0252 (4)	
H8A	0.917481	0.854469	0.810933	0.030*	
H8B	0.732289	0.882757	0.905492	0.030*	
C9	0.4511 (3)	0.6540 (2)	0.74873 (17)	0.0224 (3)	

### 4-Methoxybicyclo[3.3.1]non-3-ene-2,9-dione (1) Atomic displacement parameters (Å^2^)

**Table:**

	*U*^11^	*U*^22^	*U*^33^	*U*^12^	*U*^13^	*U*^23^
O1	0.0417 (8)	0.0189 (6)	0.0482 (8)	0.0001 (6)	0.0112 (6)	0.0026 (5)
O2	0.0288 (7)	0.0263 (6)	0.0225 (5)	−0.0041 (5)	0.0078 (4)	−0.0002 (4)
O3	0.0259 (7)	0.0372 (7)	0.0423 (7)	0.0078 (6)	0.0109 (5)	0.0059 (6)
C1	0.0235 (8)	0.0187 (8)	0.0219 (7)	0.0019 (6)	0.0033 (6)	0.0010 (6)
C2	0.0190 (7)	0.0237 (7)	0.0189 (6)	−0.0012 (6)	0.0015 (6)	0.0008 (6)
C10	0.0338 (10)	0.0371 (9)	0.0274 (8)	−0.0049 (8)	0.0139 (7)	−0.0061 (8)
C3	0.0247 (8)	0.0227 (8)	0.0250 (8)	0.0005 (6)	0.0041 (6)	−0.0039 (6)
C4	0.0222 (9)	0.0205 (8)	0.0291 (8)	−0.0004 (6)	0.0020 (6)	0.0003 (6)
C5	0.0236 (9)	0.0231 (8)	0.0264 (8)	−0.0017 (6)	0.0070 (6)	0.0041 (6)
C6	0.0348 (10)	0.0282 (9)	0.0227 (7)	−0.0008 (7)	0.0050 (6)	0.0056 (6)
C7	0.0270 (9)	0.0315 (9)	0.0230 (7)	−0.0001 (7)	−0.0007 (6)	0.0008 (7)
C8	0.0283 (9)	0.0235 (8)	0.0237 (8)	−0.0037 (7)	0.0042 (6)	−0.0030 (6)
C9	0.0236 (8)	0.0225 (7)	0.0219 (7)	0.0021 (6)	0.0057 (6)	−0.0012 (6)

### 4-Methoxybicyclo[3.3.1]non-3-ene-2,9-dione (1) Geometric parameters (Å, º)

**Table:**

O1—C4	1.225 (2)	C3—H3	0.9500
O2—C2	1.3377 (19)	C4—C5	1.529 (2)
O2—C10	1.443 (2)	C5—C9	1.505 (2)
O3—C9	1.208 (2)	C5—C6	1.554 (2)
C1—C2	1.502 (2)	C5—H5	1.0000
C1—C9	1.510 (2)	C6—C7	1.527 (3)
C1—C8	1.554 (2)	C6—H6A	0.9900
C1—H1	1.0000	C6—H6B	0.9900
C2—C3	1.351 (2)	C7—C8	1.526 (2)
C10—H10A	0.9800	C7—H7A	0.9900
C10—H10B	0.9800	C7—H7B	0.9900
C10—H10C	0.9800	C8—H8A	0.9900
C3—C4	1.451 (2)	C8—H8B	0.9900
			
C2—O2—C10	116.97 (14)	C9—C5—H5	109.7
C2—C1—C9	107.21 (13)	C4—C5—H5	109.7
C2—C1—C8	111.78 (13)	C6—C5—H5	109.7
C9—C1—C8	108.24 (12)	C7—C6—C5	111.67 (13)
C2—C1—H1	109.8	C7—C6—H6A	109.3
C9—C1—H1	109.8	C5—C6—H6A	109.3
C8—C1—H1	109.8	C7—C6—H6B	109.3
O2—C2—C3	125.52 (15)	C5—C6—H6B	109.3
O2—C2—C1	111.23 (14)	H6A—C6—H6B	107.9
C3—C2—C1	123.25 (14)	C8—C7—C6	111.97 (15)
O2—C10—H10A	109.5	C8—C7—H7A	109.2
O2—C10—H10B	109.5	C6—C7—H7A	109.2
H10A—C10—H10B	109.5	C8—C7—H7B	109.2
O2—C10—H10C	109.5	C6—C7—H7B	109.2
H10A—C10—H10C	109.5	H7A—C7—H7B	107.9
H10B—C10—H10C	109.5	C7—C8—C1	112.38 (14)
C2—C3—C4	120.89 (15)	C7—C8—H8A	109.1
C2—C3—H3	119.6	C1—C8—H8A	109.1
C4—C3—H3	119.6	C7—C8—H8B	109.1
O1—C4—C3	123.06 (16)	C1—C8—H8B	109.1
O1—C4—C5	119.00 (15)	H8A—C8—H8B	107.9
C3—C4—C5	117.94 (14)	O3—C9—C5	124.04 (16)
C9—C5—C4	110.31 (13)	O3—C9—C1	124.16 (15)
C9—C5—C6	107.41 (13)	C5—C9—C1	111.78 (14)
C4—C5—C6	110.00 (14)		
			
C10—O2—C2—C3	1.4 (2)	C9—C5—C6—C7	−57.61 (19)
C10—O2—C2—C1	−178.52 (14)	C4—C5—C6—C7	62.47 (18)
C9—C1—C2—O2	149.74 (13)	C5—C6—C7—C8	52.43 (19)
C8—C1—C2—O2	−91.79 (15)	C6—C7—C8—C1	−50.79 (18)
C9—C1—C2—C3	−30.1 (2)	C2—C1—C8—C7	−63.52 (18)
C8—C1—C2—C3	88.33 (19)	C9—C1—C8—C7	54.34 (18)
O2—C2—C3—C4	179.00 (15)	C4—C5—C9—O3	125.58 (18)
C1—C2—C3—C4	−1.1 (3)	C6—C5—C9—O3	−114.54 (18)
C2—C3—C4—O1	−174.35 (17)	C4—C5—C9—C1	−56.12 (18)
C2—C3—C4—C5	5.1 (2)	C6—C5—C9—C1	63.76 (17)
O1—C4—C5—C9	−157.28 (16)	C2—C1—C9—O3	−123.17 (17)
C3—C4—C5—C9	23.2 (2)	C8—C1—C9—O3	116.10 (18)
O1—C4—C5—C6	84.41 (19)	C2—C1—C9—C5	58.54 (16)
C3—C4—C5—C6	−95.07 (17)	C8—C1—C9—C5	−62.20 (17)

### 4,9,9-Trimethoxybicyclo[3.3.1]non-3-en-2-ol (2) Crystal data

**Table:**

C_12_H_20_O_4_	*Z* = 4
*M**_r_* = 228.28	*F*(000) = 496
Triclinic, *P*1	*D*_x_ = 1.317 Mg m^−^^3^
*a* = 7.7330 (15) Å	Mo *K*α radiation, λ = 0.71073 Å
*b* = 10.976 (2) Å	Cell parameters from 1376 reflections
*c* = 13.874 (3) Å	θ = 3.0–25.0°
α = 101.984 (5)°	µ = 0.10 mm^−^^1^
β = 92.032 (5)°	*T* = 152 K
γ = 90.293 (5)°	Plate, colourless
*V* = 1151.2 (4) Å^3^	0.28 × 0.24 × 0.04 mm

### 4,9,9-Trimethoxybicyclo[3.3.1]non-3-en-2-ol (2) Data collection

**Table:**

Bruker D8 VENTURE PHOTON II diffractometer	2677 reflections with *I* > 2σ(*I*)
Radiation source: INCOATEC IÂµS microfocus sealed tube	*R*_int_ = 0.055
φ and ω scans	θ_max_ = 26.4°, θ_min_ = 1.5°
Absorption correction: multi-scan (SADABS; Krause *et al.*, 2015)	*h* = −9→9
*T*_min_ = 0.578, *T*_max_ = 0.746	*k* = −13→13
8885 measured reflections	*l* = −17→17
4662 independent reflections	

### 4,9,9-Trimethoxybicyclo[3.3.1]non-3-en-2-ol (2) Refinement

**Table:**

Refinement on *F*^2^	Primary atom site location: structure-invariant direct methods
Least-squares matrix: full	Secondary atom site location: difference Fourier map
*R*[*F*^2^ > 2σ(*F*^2^)] = 0.057	Hydrogen site location: mixed
*wR*(*F*^2^) = 0.139	H atoms treated by a mixture of independent and constrained refinement
*S* = 0.98	*w* = 1/[σ^2^(*F*_o_^2^) + (0.0513*P*)^2^] where *P* = (*F*_o_^2^ + 2*F*_c_^2^)/3
4662 reflections	(Δ/σ)_max_ < 0.001
301 parameters	Δρ_max_ = 0.24 e Å^−^^3^
2 restraints	Δρ_min_ = −0.24 e Å^−^^3^

### 4,9,9-Trimethoxybicyclo[3.3.1]non-3-en-2-ol (2) Special details

**Table:**

Geometry. All esds (except the esd in the dihedral angle between two l.s. planes) are estimated using the full covariance matrix. The cell esds are taken into account individually in the estimation of esds in distances, angles and torsion angles; correlations between esds in cell parameters are only used when they are defined by crystal symmetry. An approximate (isotropic) treatment of cell esds is used for estimating esds involving l.s. planes.

### 4,9,9-Trimethoxybicyclo[3.3.1]non-3-en-2-ol (2) Fractional atomic coordinates and isotropic or equivalent isotropic displacement parameters (Å^2^)

**Table:**

	*x*	*y*	*z*	*U*_iso_*/*U*_eq_	
O3	0.2250 (2)	0.37070 (16)	0.13978 (14)	0.0244 (5)	
O2	0.7476 (2)	0.61301 (17)	0.27191 (16)	0.0288 (5)	
H2O	0.741 (4)	0.6909 (10)	0.278 (2)	0.043*	
O1	0.5543 (2)	0.27872 (16)	0.40336 (14)	0.0248 (5)	
O4	0.2372 (2)	0.50789 (16)	0.29210 (14)	0.0219 (4)	
O5	1.0600 (2)	1.16441 (16)	0.39368 (14)	0.0252 (5)	
O6	1.2486 (2)	0.75906 (17)	0.26446 (16)	0.0311 (5)	
H6O	1.234 (4)	0.6854 (14)	0.271 (2)	0.047*	
O7	0.7194 (2)	0.92784 (17)	0.13891 (14)	0.0252 (5)	
O8	0.7424 (2)	0.87754 (16)	0.29317 (13)	0.0208 (4)	
C1	0.4127 (3)	0.3167 (2)	0.2597 (2)	0.0207 (6)	
H1	0.317759	0.267373	0.281279	0.025*	
C2	0.5345 (3)	0.3652 (2)	0.3458 (2)	0.0206 (6)	
C3	0.6152 (3)	0.4749 (2)	0.3592 (2)	0.0211 (6)	
H3	0.695548	0.498054	0.413456	0.025*	
C4	0.5835 (3)	0.5635 (2)	0.2916 (2)	0.0210 (6)	
H4	0.514429	0.634247	0.327706	0.025*	
C5	0.4824 (3)	0.5061 (2)	0.1964 (2)	0.0213 (6)	
H5	0.430621	0.575362	0.168691	0.026*	
C6	0.5910 (3)	0.4263 (3)	0.1161 (2)	0.0263 (7)	
H6A	0.520091	0.406174	0.053892	0.032*	
H6B	0.692026	0.476413	0.104437	0.032*	
C7	0.6560 (3)	0.3048 (3)	0.1411 (2)	0.0276 (7)	
H7A	0.749392	0.323841	0.192827	0.033*	
H7B	0.705050	0.252400	0.081777	0.033*	
C8	0.5107 (3)	0.2326 (2)	0.1773 (2)	0.0244 (7)	
H8A	0.560293	0.161608	0.202263	0.029*	
H8B	0.428924	0.198770	0.121522	0.029*	
C9	0.3347 (3)	0.4262 (2)	0.2210 (2)	0.0200 (6)	
C10	0.1260 (4)	0.4544 (3)	0.0941 (2)	0.0324 (7)	
H10A	0.054707	0.406678	0.039144	0.049*	
H10B	0.204374	0.510236	0.069224	0.049*	
H10C	0.051003	0.503695	0.142526	0.049*	
C11	0.0995 (3)	0.4525 (3)	0.3350 (2)	0.0306 (7)	
H11A	0.019317	0.517320	0.364698	0.046*	
H11B	0.147237	0.411031	0.385985	0.046*	
H11C	0.037487	0.391278	0.283835	0.046*	
C12	0.6804 (4)	0.3079 (3)	0.4830 (2)	0.0311 (7)	
H12A	0.682747	0.240840	0.519879	0.047*	
H12B	0.650051	0.386227	0.526962	0.047*	
H12C	0.794670	0.316700	0.456675	0.047*	
C13	0.9185 (3)	1.0473 (2)	0.2509 (2)	0.0212 (6)	
H13	0.826248	1.109938	0.271311	0.025*	
C14	1.0410 (3)	1.0473 (2)	0.3369 (2)	0.0203 (6)	
C15	1.1210 (3)	0.9454 (2)	0.3512 (2)	0.0200 (6)	
H15	1.203910	0.952686	0.404336	0.024*	
C16	1.0862 (3)	0.8197 (2)	0.2871 (2)	0.0225 (6)	
H16	1.019848	0.769528	0.326094	0.027*	
C17	0.9785 (3)	0.8221 (2)	0.1917 (2)	0.0214 (6)	
H17	0.923065	0.738204	0.168204	0.026*	
C18	1.0814 (3)	0.8527 (2)	0.1075 (2)	0.0268 (7)	
H18A	1.005550	0.839472	0.047137	0.032*	
H18B	1.178675	0.793939	0.094485	0.032*	
C19	1.1541 (3)	0.9861 (3)	0.1278 (2)	0.0281 (7)	
H19A	1.249799	0.994633	0.178169	0.034*	
H19B	1.201510	1.003598	0.066630	0.034*	
C20	1.0141 (3)	1.0806 (3)	0.1641 (2)	0.0264 (7)	
H20A	0.929714	1.083264	0.109210	0.032*	
H20B	1.067692	1.164458	0.185194	0.032*	
C21	0.8344 (3)	0.9177 (2)	0.2176 (2)	0.0198 (6)	
C22	0.6196 (4)	0.8179 (3)	0.0962 (2)	0.0347 (8)	
H22A	0.531624	0.838293	0.049607	0.052*	
H22B	0.563015	0.786549	0.148393	0.052*	
H22C	0.696186	0.754021	0.061296	0.052*	
C23	0.6060 (3)	0.9566 (3)	0.3360 (2)	0.0288 (7)	
H23A	0.535246	0.912043	0.375069	0.043*	
H23B	0.533485	0.979948	0.283521	0.043*	
H23C	0.656028	1.031866	0.378740	0.043*	
C24	1.1744 (4)	1.1781 (3)	0.4781 (2)	0.0305 (7)	
H24A	1.172238	1.264278	0.515319	0.046*	
H24B	1.292245	1.157427	0.457040	0.046*	
H24C	1.137482	1.121833	0.520074	0.046*	

### 4,9,9-Trimethoxybicyclo[3.3.1]non-3-en-2-ol (2) Atomic displacement parameters (Å^2^)

**Table:**

	*U*^11^	*U*^22^	*U*^33^	*U*^12^	*U*^13^	*U*^23^
O3	0.0254 (10)	0.0188 (10)	0.0306 (12)	0.0042 (7)	−0.0063 (9)	0.0099 (9)
O2	0.0218 (10)	0.0161 (10)	0.0522 (15)	−0.0008 (8)	0.0037 (9)	0.0149 (10)
O1	0.0323 (10)	0.0170 (10)	0.0288 (12)	0.0030 (8)	−0.0047 (9)	0.0141 (9)
O4	0.0210 (9)	0.0145 (10)	0.0318 (12)	0.0038 (7)	0.0054 (8)	0.0081 (8)
O5	0.0311 (10)	0.0146 (10)	0.0297 (12)	0.0001 (8)	−0.0046 (9)	0.0052 (9)
O6	0.0242 (10)	0.0181 (11)	0.0546 (15)	0.0102 (8)	0.0080 (10)	0.0145 (10)
O7	0.0263 (10)	0.0212 (11)	0.0296 (12)	−0.0021 (8)	−0.0062 (9)	0.0102 (9)
O8	0.0200 (9)	0.0168 (10)	0.0278 (12)	0.0046 (7)	0.0033 (8)	0.0091 (8)
C1	0.0223 (13)	0.0135 (14)	0.0289 (17)	0.0014 (10)	−0.0007 (12)	0.0106 (12)
C2	0.0225 (13)	0.0151 (14)	0.0271 (17)	0.0069 (10)	0.0043 (12)	0.0105 (12)
C3	0.0203 (13)	0.0194 (15)	0.0260 (17)	0.0031 (11)	−0.0013 (12)	0.0102 (12)
C4	0.0192 (13)	0.0148 (14)	0.0310 (17)	0.0022 (10)	0.0051 (12)	0.0086 (12)
C5	0.0210 (13)	0.0144 (14)	0.0320 (17)	0.0066 (10)	0.0036 (12)	0.0119 (12)
C6	0.0291 (15)	0.0262 (16)	0.0268 (17)	0.0032 (12)	0.0048 (13)	0.0120 (14)
C7	0.0271 (15)	0.0246 (16)	0.0310 (18)	0.0077 (12)	0.0030 (13)	0.0047 (14)
C8	0.0290 (15)	0.0155 (14)	0.0289 (18)	0.0075 (11)	−0.0036 (13)	0.0058 (13)
C9	0.0182 (13)	0.0169 (14)	0.0259 (16)	0.0028 (10)	0.0005 (12)	0.0071 (12)
C10	0.0336 (16)	0.0265 (17)	0.040 (2)	0.0082 (13)	−0.0095 (14)	0.0160 (15)
C11	0.0243 (14)	0.0288 (17)	0.043 (2)	0.0037 (12)	0.0103 (14)	0.0154 (15)
C12	0.0379 (17)	0.0286 (17)	0.0294 (18)	0.0076 (13)	−0.0025 (14)	0.0126 (14)
C13	0.0237 (13)	0.0108 (13)	0.0313 (17)	0.0035 (10)	−0.0031 (12)	0.0100 (12)
C14	0.0224 (13)	0.0147 (14)	0.0248 (16)	−0.0008 (10)	0.0024 (12)	0.0062 (12)
C15	0.0194 (13)	0.0186 (14)	0.0236 (16)	0.0027 (10)	−0.0010 (11)	0.0083 (12)
C16	0.0179 (13)	0.0152 (14)	0.0380 (18)	0.0080 (10)	0.0057 (12)	0.0127 (13)
C17	0.0213 (13)	0.0129 (13)	0.0310 (17)	0.0015 (10)	0.0012 (12)	0.0071 (12)
C18	0.0304 (15)	0.0226 (16)	0.0283 (18)	0.0014 (12)	0.0082 (13)	0.0063 (13)
C19	0.0311 (15)	0.0286 (17)	0.0273 (18)	−0.0036 (12)	0.0046 (13)	0.0118 (14)
C20	0.0258 (14)	0.0260 (16)	0.0309 (18)	−0.0042 (12)	−0.0027 (13)	0.0150 (14)
C21	0.0201 (13)	0.0166 (14)	0.0242 (16)	0.0006 (10)	−0.0015 (12)	0.0084 (12)
C22	0.0385 (17)	0.0254 (17)	0.039 (2)	−0.0050 (13)	−0.0139 (15)	0.0060 (15)
C23	0.0255 (15)	0.0244 (16)	0.040 (2)	0.0108 (11)	0.0106 (13)	0.0121 (14)
C24	0.0343 (16)	0.0291 (17)	0.0285 (18)	−0.0054 (13)	−0.0017 (14)	0.0079 (14)

### 4,9,9-Trimethoxybicyclo[3.3.1]non-3-en-2-ol (2) Geometric parameters (Å, º)

**Table:**

O3—C9	1.415 (3)	C10—H10B	0.9800
O3—C10	1.431 (3)	C10—H10C	0.9800
O2—C4	1.435 (3)	C11—H11A	0.9800
O2—H2O	0.843 (10)	C11—H11B	0.9800
O1—C2	1.367 (3)	C11—H11C	0.9800
O1—C12	1.431 (3)	C12—H12A	0.9800
O4—C9	1.427 (3)	C12—H12B	0.9800
O4—C11	1.431 (3)	C12—H12C	0.9800
O5—C14	1.365 (3)	C13—C14	1.497 (4)
O5—C24	1.425 (3)	C13—C21	1.536 (3)
O6—C16	1.438 (3)	C13—C20	1.541 (4)
O6—H6O	0.842 (10)	C13—H13	1.0000
O7—C21	1.406 (3)	C14—C15	1.328 (3)
O7—C22	1.436 (3)	C15—C16	1.496 (4)
O8—C21	1.429 (3)	C15—H15	0.9500
O8—C23	1.435 (3)	C16—C17	1.543 (4)
C1—C2	1.499 (4)	C16—H16	1.0000
C1—C9	1.533 (3)	C17—C18	1.529 (3)
C1—C8	1.538 (4)	C17—C21	1.534 (3)
C1—H1	1.0000	C17—H17	1.0000
C2—C3	1.329 (3)	C18—C19	1.533 (4)
C3—C4	1.500 (3)	C18—H18A	0.9900
C3—H3	0.9500	C18—H18B	0.9900
C4—C5	1.526 (4)	C19—C20	1.528 (4)
C4—H4	1.0000	C19—H19A	0.9900
C5—C9	1.528 (3)	C19—H19B	0.9900
C5—C6	1.543 (4)	C20—H20A	0.9900
C5—H5	1.0000	C20—H20B	0.9900
C6—C7	1.529 (4)	C22—H22A	0.9800
C6—H6A	0.9900	C22—H22B	0.9800
C6—H6B	0.9900	C22—H22C	0.9800
C7—C8	1.529 (4)	C23—H23A	0.9800
C7—H7A	0.9900	C23—H23B	0.9800
C7—H7B	0.9900	C23—H23C	0.9800
C8—H8A	0.9900	C24—H24A	0.9800
C8—H8B	0.9900	C24—H24B	0.9800
C10—H10A	0.9800	C24—H24C	0.9800
			
C9—O3—C10	116.1 (2)	H12A—C12—H12B	109.5
C4—O2—H2O	110 (2)	O1—C12—H12C	109.5
C2—O1—C12	116.2 (2)	H12A—C12—H12C	109.5
C9—O4—C11	116.6 (2)	H12B—C12—H12C	109.5
C14—O5—C24	116.6 (2)	C14—C13—C21	109.5 (2)
C16—O6—H6O	106 (2)	C14—C13—C20	110.8 (2)
C21—O7—C22	116.0 (2)	C21—C13—C20	109.1 (2)
C21—O8—C23	116.4 (2)	C14—C13—H13	109.1
C2—C1—C9	109.6 (2)	C21—C13—H13	109.1
C2—C1—C8	109.8 (2)	C20—C13—H13	109.1
C9—C1—C8	109.5 (2)	C15—C14—O5	126.7 (2)
C2—C1—H1	109.3	C15—C14—C13	122.6 (2)
C9—C1—H1	109.3	O5—C14—C13	110.7 (2)
C8—C1—H1	109.3	C14—C15—C16	122.8 (2)
C3—C2—O1	126.4 (2)	C14—C15—H15	118.6
C3—C2—C1	123.2 (2)	C16—C15—H15	118.6
O1—C2—C1	110.3 (2)	O6—C16—C15	108.7 (2)
C2—C3—C4	122.2 (2)	O6—C16—C17	110.7 (2)
C2—C3—H3	118.9	C15—C16—C17	114.2 (2)
C4—C3—H3	118.9	O6—C16—H16	107.7
O2—C4—C3	108.1 (2)	C15—C16—H16	107.7
O2—C4—C5	111.5 (2)	C17—C16—H16	107.7
C3—C4—C5	113.9 (2)	C18—C17—C21	109.5 (2)
O2—C4—H4	107.7	C18—C17—C16	114.9 (2)
C3—C4—H4	107.7	C21—C17—C16	108.0 (2)
C5—C4—H4	107.7	C18—C17—H17	108.1
C4—C5—C9	108.7 (2)	C21—C17—H17	108.1
C4—C5—C6	114.8 (2)	C16—C17—H17	108.1
C9—C5—C6	109.0 (2)	C17—C18—C19	114.0 (2)
C4—C5—H5	108.0	C17—C18—H18A	108.8
C9—C5—H5	108.0	C19—C18—H18A	108.8
C6—C5—H5	108.0	C17—C18—H18B	108.8
C7—C6—C5	114.4 (2)	C19—C18—H18B	108.8
C7—C6—H6A	108.7	H18A—C18—H18B	107.6
C5—C6—H6A	108.7	C20—C19—C18	111.1 (2)
C7—C6—H6B	108.7	C20—C19—H19A	109.4
C5—C6—H6B	108.7	C18—C19—H19A	109.4
H6A—C6—H6B	107.6	C20—C19—H19B	109.4
C6—C7—C8	111.6 (2)	C18—C19—H19B	109.4
C6—C7—H7A	109.3	H19A—C19—H19B	108.0
C8—C7—H7A	109.3	C19—C20—C13	111.6 (2)
C6—C7—H7B	109.3	C19—C20—H20A	109.3
C8—C7—H7B	109.3	C13—C20—H20A	109.3
H7A—C7—H7B	108.0	C19—C20—H20B	109.3
C7—C8—C1	111.1 (2)	C13—C20—H20B	109.3
C7—C8—H8A	109.4	H20A—C20—H20B	108.0
C1—C8—H8A	109.4	O7—C21—O8	109.84 (19)
C7—C8—H8B	109.4	O7—C21—C17	115.2 (2)
C1—C8—H8B	109.4	O8—C21—C17	104.9 (2)
H8A—C8—H8B	108.0	O7—C21—C13	105.2 (2)
O3—C9—O4	109.76 (19)	O8—C21—C13	113.6 (2)
O3—C9—C5	115.0 (2)	C17—C21—C13	108.3 (2)
O4—C9—C5	105.4 (2)	O7—C22—H22A	109.5
O3—C9—C1	105.0 (2)	O7—C22—H22B	109.5
O4—C9—C1	113.5 (2)	H22A—C22—H22B	109.5
C5—C9—C1	108.5 (2)	O7—C22—H22C	109.5
O3—C10—H10A	109.5	H22A—C22—H22C	109.5
O3—C10—H10B	109.5	H22B—C22—H22C	109.5
H10A—C10—H10B	109.5	O8—C23—H23A	109.5
O3—C10—H10C	109.5	O8—C23—H23B	109.5
H10A—C10—H10C	109.5	H23A—C23—H23B	109.5
H10B—C10—H10C	109.5	O8—C23—H23C	109.5
O4—C11—H11A	109.5	H23A—C23—H23C	109.5
O4—C11—H11B	109.5	H23B—C23—H23C	109.5
H11A—C11—H11B	109.5	O5—C24—H24A	109.5
O4—C11—H11C	109.5	O5—C24—H24B	109.5
H11A—C11—H11C	109.5	H24A—C24—H24B	109.5
H11B—C11—H11C	109.5	O5—C24—H24C	109.5
O1—C12—H12A	109.5	H24A—C24—H24C	109.5
O1—C12—H12B	109.5	H24B—C24—H24C	109.5
			
C12—O1—C2—C3	−3.0 (4)	C24—O5—C14—C15	−1.5 (4)
C12—O1—C2—C1	174.1 (2)	C24—O5—C14—C13	−179.6 (2)
C9—C1—C2—C3	−25.6 (4)	C21—C13—C14—C15	27.9 (4)
C8—C1—C2—C3	94.7 (3)	C20—C13—C14—C15	−92.4 (3)
C9—C1—C2—O1	157.2 (2)	C21—C13—C14—O5	−153.9 (2)
C8—C1—C2—O1	−82.5 (3)	C20—C13—C14—O5	85.8 (3)
O1—C2—C3—C4	−179.8 (2)	O5—C14—C15—C16	177.4 (2)
C1—C2—C3—C4	3.4 (4)	C13—C14—C15—C16	−4.7 (4)
C2—C3—C4—O2	−136.6 (3)	C14—C15—C16—O6	135.7 (3)
C2—C3—C4—C5	−12.2 (4)	C14—C15—C16—C17	11.7 (4)
O2—C4—C5—C9	165.22 (19)	O6—C16—C17—C18	−41.7 (3)
C3—C4—C5—C9	42.6 (3)	C15—C16—C17—C18	81.3 (3)
O2—C4—C5—C6	42.8 (3)	O6—C16—C17—C21	−164.30 (19)
C3—C4—C5—C6	−79.8 (3)	C15—C16—C17—C21	−41.3 (3)
C4—C5—C6—C7	68.5 (3)	C21—C17—C18—C19	54.7 (3)
C9—C5—C6—C7	−53.7 (3)	C16—C17—C18—C19	−67.0 (3)
C5—C6—C7—C8	48.6 (3)	C17—C18—C19—C20	−49.6 (3)
C6—C7—C8—C1	−50.6 (3)	C18—C19—C20—C13	51.3 (3)
C2—C1—C8—C7	−60.8 (3)	C14—C13—C20—C19	61.2 (3)
C9—C1—C8—C7	59.5 (3)	C21—C13—C20—C19	−59.4 (3)
C10—O3—C9—O4	53.5 (3)	C22—O7—C21—O8	−55.6 (3)
C10—O3—C9—C5	−65.1 (3)	C22—O7—C21—C17	62.5 (3)
C10—O3—C9—C1	175.8 (2)	C22—O7—C21—C13	−178.3 (2)
C11—O4—C9—O3	61.6 (3)	C23—O8—C21—O7	−59.8 (3)
C11—O4—C9—C5	−174.1 (2)	C23—O8—C21—C17	175.9 (2)
C11—O4—C9—C1	−55.6 (3)	C23—O8—C21—C13	57.7 (3)
C4—C5—C9—O3	177.56 (19)	C18—C17—C21—O7	56.6 (3)
C6—C5—C9—O3	−56.6 (3)	C16—C17—C21—O7	−177.58 (19)
C4—C5—C9—O4	56.6 (2)	C18—C17—C21—O8	177.5 (2)
C6—C5—C9—O4	−177.5 (2)	C16—C17—C21—O8	−56.7 (2)
C4—C5—C9—C1	−65.2 (3)	C18—C17—C21—C13	−60.9 (3)
C6—C5—C9—C1	60.6 (3)	C16—C17—C21—C13	64.9 (3)
C2—C1—C9—O3	179.1 (2)	C14—C13—C21—O7	178.6 (2)
C8—C1—C9—O3	58.6 (2)	C20—C13—C21—O7	−60.0 (3)
C2—C1—C9—O4	−61.0 (3)	C14—C13—C21—O8	58.4 (3)
C8—C1—C9—O4	178.5 (2)	C20—C13—C21—O8	179.81 (19)
C2—C1—C9—C5	55.7 (3)	C14—C13—C21—C17	−57.7 (3)
C8—C1—C9—C5	−64.8 (3)	C20—C13—C21—C17	63.7 (3)

### 4,9,9-Trimethoxybicyclo[3.3.1]non-3-en-2-ol (2) Hydrogen-bond geometry (Å, º)

**Table:**

*D*—H···*A*	*D*—H	H···*A*	*D*···*A*	*D*—H···*A*
O6—H6*O*···O4^i^	0.84 (1)	2.03 (1)	2.863 (3)	172 (3)
O2—H2*O*···O8	0.84 (1)	2.02 (1)	2.858 (3)	176 (3)

Symmetry code: (i) *x*+1, *y*, *z*.

### 4-Methoxy-6-methyl-1-(3-methylbut-2-en-1-yl)-6-(4-methylpent-3-en-1-yl)bicyclo[3.3.1]non-3-ene-2,9-dione (3) Crystal data

**Table:**

C_22_H_32_O_3_	*F*(000) = 752
*M**_r_* = 344.47	*D*_x_ = 1.161 Mg m^−^^3^
Monoclinic, *P*2_1_/*n*	Mo *K*α radiation, λ = 0.71073 Å
*a* = 14.1871 (6) Å	Cell parameters from 5403 reflections
*b* = 6.3306 (2) Å	θ = 2.9–27.0°
*c* = 22.4159 (8) Å	µ = 0.08 mm^−^^1^
β = 101.702 (1)°	*T* = 133 K
*V* = 1971.39 (13) Å^3^	Plate, colourless
*Z* = 4	0.18 × 0.14 × 0.04 mm

### 4-Methoxy-6-methyl-1-(3-methylbut-2-en-1-yl)-6-(4-methylpent-3-en-1-yl)bicyclo[3.3.1]non-3-ene-2,9-dione (3) Data collection

**Table:**

Bruker D8 VENTURE PHOTON II diffractometer	3271 reflections with *I* > 2σ(*I*)
Radiation source: INCOATEC IÂµS microfocus sealed tube	*R*_int_ = 0.067
φ and ω scans	θ_max_ = 27.1°, θ_min_ = 2.9°
Absorption correction: multi-scan (SADABS; Krause *et al.*, 2015)	*h* = −18→18
*T*_min_ = 0.687, *T*_max_ = 0.746	*k* = −8→7
28995 measured reflections	*l* = −28→28
4348 independent reflections	

### 4-Methoxy-6-methyl-1-(3-methylbut-2-en-1-yl)-6-(4-methylpent-3-en-1-yl)bicyclo[3.3.1]non-3-ene-2,9-dione (3) Refinement

**Table:**

Refinement on *F*^2^	Primary atom site location: structure-invariant direct methods
Least-squares matrix: full	Secondary atom site location: difference Fourier map
*R*[*F*^2^ > 2σ(*F*^2^)] = 0.046	Hydrogen site location: inferred from neighbouring sites
*wR*(*F*^2^) = 0.116	H-atom parameters constrained
*S* = 1.05	*w* = 1/[σ^2^(*F*_o_^2^) + (0.0448*P*)^2^ + 0.8443*P*] where *P* = (*F*_o_^2^ + 2*F*_c_^2^)/3
4348 reflections	(Δ/σ)_max_ = 0.001
232 parameters	Δρ_max_ = 0.28 e Å^−^^3^
0 restraints	Δρ_min_ = −0.22 e Å^−^^3^

### 4-Methoxy-6-methyl-1-(3-methylbut-2-en-1-yl)-6-(4-methylpent-3-en-1-yl)bicyclo[3.3.1]non-3-ene-2,9-dione (3) Special details

**Table:**

Geometry. All esds (except the esd in the dihedral angle between two l.s. planes) are estimated using the full covariance matrix. The cell esds are taken into account individually in the estimation of esds in distances, angles and torsion angles; correlations between esds in cell parameters are only used when they are defined by crystal symmetry. An approximate (isotropic) treatment of cell esds is used for estimating esds involving l.s. planes.

### 4-Methoxy-6-methyl-1-(3-methylbut-2-en-1-yl)-6-(4-methylpent-3-en-1-yl)bicyclo[3.3.1]non-3-ene-2,9-dione (3) Fractional atomic coordinates and isotropic or equivalent isotropic displacement parameters (Å^2^)

**Table:**

	*x*	*y*	*z*	*U*_iso_*/*U*_eq_	
O1	0.39938 (7)	0.15883 (17)	0.60348 (5)	0.0219 (2)	
O2	0.65032 (8)	0.42454 (18)	0.51454 (5)	0.0248 (3)	
O3	0.69009 (8)	0.20509 (19)	0.71052 (5)	0.0270 (3)	
C1	0.53136 (10)	0.3264 (2)	0.66586 (7)	0.0180 (3)	
H1	0.511136	0.232666	0.696903	0.022*	
C2	0.48471 (10)	0.2511 (2)	0.60337 (7)	0.0177 (3)	
C11	0.81757 (10)	0.2004 (3)	0.60868 (7)	0.0212 (3)	
H11	0.807199	0.084368	0.633448	0.025*	
C12	0.85993 (11)	0.1573 (3)	0.56230 (7)	0.0223 (3)	
C13	0.89729 (12)	−0.0610 (3)	0.55400 (8)	0.0299 (4)	
H13A	0.865158	−0.116923	0.514308	0.045*	
H13B	0.884177	−0.153610	0.586396	0.045*	
H13C	0.966851	−0.054570	0.555907	0.045*	
C14	0.87732 (13)	0.3143 (3)	0.51526 (8)	0.0310 (4)	
H14A	0.848722	0.261744	0.474485	0.047*	
H14B	0.946743	0.333557	0.518615	0.047*	
H14C	0.847832	0.449848	0.522063	0.047*	
C15	0.55095 (12)	0.6122 (3)	0.74496 (7)	0.0265 (4)	
H15A	0.532511	0.755107	0.754900	0.040*	
H15B	0.621187	0.603420	0.750744	0.040*	
H15C	0.528252	0.510118	0.771758	0.040*	
C16	0.39580 (11)	0.5993 (3)	0.66583 (7)	0.0218 (3)	
H16A	0.384466	0.751796	0.671266	0.026*	
H16B	0.369708	0.565027	0.622540	0.026*	
C17	0.33786 (11)	0.4754 (3)	0.70482 (7)	0.0257 (4)	
H17A	0.339038	0.323133	0.694948	0.031*	
H17B	0.367411	0.493600	0.748477	0.031*	
C18	0.23549 (11)	0.5523 (3)	0.69309 (8)	0.0279 (4)	
H18	0.225678	0.687173	0.709382	0.033*	
C19	0.15695 (12)	0.4559 (3)	0.66287 (8)	0.0268 (4)	
C20	0.15578 (14)	0.2407 (3)	0.63505 (9)	0.0399 (5)	
H20A	0.104596	0.155924	0.646702	0.060*	
H20B	0.218028	0.171697	0.649642	0.060*	
H20C	0.143955	0.253524	0.590581	0.060*	
C21	0.06030 (12)	0.5629 (3)	0.65247 (9)	0.0378 (5)	
H21A	0.015217	0.476108	0.669694	0.057*	
H21B	0.035856	0.580812	0.608633	0.057*	
H21C	0.066863	0.701625	0.672309	0.057*	
C22	0.34621 (11)	0.0763 (3)	0.54652 (8)	0.0271 (4)	
H22A	0.288167	0.004550	0.553438	0.041*	
H22B	0.386501	−0.024249	0.529772	0.041*	
H22C	0.327832	0.192700	0.517674	0.041*	
C3	0.52562 (10)	0.2750 (2)	0.55459 (7)	0.0202 (3)	
H3	0.493355	0.222681	0.516124	0.024*	
C4	0.61796 (11)	0.3789 (2)	0.55963 (7)	0.0190 (3)	
C5	0.67586 (10)	0.4359 (2)	0.62303 (7)	0.0180 (3)	
C6	0.65342 (11)	0.6707 (2)	0.63564 (7)	0.0218 (3)	
H6A	0.692482	0.713146	0.675594	0.026*	
H6B	0.672651	0.761032	0.604069	0.026*	
C7	0.54739 (11)	0.7092 (2)	0.63586 (7)	0.0220 (3)	
H7A	0.509788	0.691352	0.593857	0.026*	
H7B	0.539427	0.857263	0.648144	0.026*	
C8	0.50547 (10)	0.5620 (2)	0.67851 (7)	0.0191 (3)	
C9	0.63940 (10)	0.3078 (2)	0.67122 (7)	0.0182 (3)	
C10	0.78446 (11)	0.4122 (3)	0.62607 (7)	0.0222 (3)	
H10A	0.804318	0.519739	0.599017	0.027*	
H10B	0.818498	0.444174	0.668169	0.027*	

### 4-Methoxy-6-methyl-1-(3-methylbut-2-en-1-yl)-6-(4-methylpent-3-en-1-yl)bicyclo[3.3.1]non-3-ene-2,9-dione (3) Atomic displacement parameters (Å^2^)

**Table:**

	*U*^11^	*U*^22^	*U*^33^	*U*^12^	*U*^13^	*U*^23^
O1	0.0186 (5)	0.0217 (6)	0.0243 (6)	−0.0033 (4)	0.0018 (4)	−0.0049 (5)
O2	0.0266 (6)	0.0293 (6)	0.0191 (6)	0.0028 (5)	0.0061 (5)	0.0040 (5)
O3	0.0232 (6)	0.0330 (7)	0.0229 (6)	0.0051 (5)	0.0002 (5)	0.0084 (5)
C1	0.0191 (7)	0.0180 (7)	0.0164 (7)	0.0000 (6)	0.0026 (6)	0.0009 (6)
C2	0.0160 (7)	0.0134 (7)	0.0221 (7)	0.0018 (5)	0.0000 (6)	0.0001 (6)
C11	0.0162 (7)	0.0226 (8)	0.0235 (8)	−0.0006 (6)	0.0007 (6)	0.0025 (6)
C12	0.0171 (7)	0.0229 (8)	0.0253 (8)	0.0003 (6)	0.0007 (6)	−0.0007 (6)
C13	0.0256 (8)	0.0272 (9)	0.0359 (10)	0.0047 (7)	0.0039 (7)	−0.0028 (8)
C14	0.0327 (9)	0.0318 (10)	0.0308 (9)	0.0006 (7)	0.0119 (7)	0.0016 (8)
C15	0.0246 (8)	0.0327 (9)	0.0213 (8)	−0.0013 (7)	0.0022 (6)	−0.0069 (7)
C16	0.0208 (8)	0.0222 (8)	0.0219 (8)	0.0032 (6)	0.0031 (6)	−0.0022 (6)
C17	0.0224 (8)	0.0319 (9)	0.0231 (8)	0.0017 (7)	0.0050 (6)	−0.0011 (7)
C18	0.0246 (8)	0.0313 (9)	0.0291 (9)	0.0026 (7)	0.0087 (7)	−0.0041 (7)
C19	0.0254 (8)	0.0319 (10)	0.0235 (8)	0.0005 (7)	0.0060 (7)	0.0035 (7)
C20	0.0359 (10)	0.0408 (12)	0.0402 (11)	−0.0035 (9)	0.0009 (8)	−0.0066 (9)
C21	0.0230 (9)	0.0478 (12)	0.0417 (11)	0.0009 (8)	0.0048 (8)	0.0060 (9)
C22	0.0212 (8)	0.0291 (9)	0.0281 (9)	−0.0046 (7)	−0.0017 (7)	−0.0087 (7)
C3	0.0205 (7)	0.0212 (8)	0.0170 (7)	0.0018 (6)	−0.0008 (6)	−0.0037 (6)
C4	0.0203 (7)	0.0165 (7)	0.0194 (8)	0.0054 (6)	0.0022 (6)	0.0013 (6)
C5	0.0172 (7)	0.0185 (8)	0.0180 (7)	−0.0003 (6)	0.0025 (6)	−0.0006 (6)
C6	0.0241 (8)	0.0176 (8)	0.0245 (8)	−0.0014 (6)	0.0065 (6)	−0.0012 (6)
C7	0.0242 (8)	0.0159 (7)	0.0262 (8)	0.0026 (6)	0.0058 (6)	−0.0014 (6)
C8	0.0198 (7)	0.0201 (8)	0.0170 (7)	0.0013 (6)	0.0025 (6)	−0.0025 (6)
C9	0.0207 (7)	0.0163 (7)	0.0161 (7)	0.0005 (6)	0.0003 (6)	−0.0018 (6)
C10	0.0183 (7)	0.0236 (8)	0.0242 (8)	−0.0015 (6)	0.0035 (6)	−0.0002 (6)

### 4-Methoxy-6-methyl-1-(3-methylbut-2-en-1-yl)-6-(4-methylpent-3-en-1-yl)bicyclo[3.3.1]non-3-ene-2,9-dione (3) Geometric parameters (Å, º)

**Table:**

O1—C2	1.3445 (17)	C17—H17A	0.9900
O1—C22	1.4425 (19)	C17—H17B	0.9900
O2—C4	1.2263 (18)	C18—C19	1.329 (2)
O3—C9	1.2078 (18)	C18—H18	0.9500
C1—C2	1.501 (2)	C19—C20	1.497 (3)
C1—C9	1.518 (2)	C19—C21	1.505 (2)
C1—C8	1.575 (2)	C20—H20A	0.9800
C1—H1	1.0000	C20—H20B	0.9800
C2—C3	1.346 (2)	C20—H20C	0.9800
C11—C12	1.331 (2)	C21—H21A	0.9800
C11—C10	1.498 (2)	C21—H21B	0.9800
C11—H11	0.9500	C21—H21C	0.9800
C12—C13	1.505 (2)	C22—H22A	0.9800
C12—C14	1.505 (2)	C22—H22B	0.9800
C13—H13A	0.9800	C22—H22C	0.9800
C13—H13B	0.9800	C3—C4	1.450 (2)
C13—H13C	0.9800	C3—H3	0.9500
C14—H14A	0.9800	C4—C5	1.533 (2)
C14—H14B	0.9800	C5—C9	1.523 (2)
C14—H14C	0.9800	C5—C10	1.536 (2)
C15—C8	1.532 (2)	C5—C6	1.558 (2)
C15—H15A	0.9800	C6—C7	1.525 (2)
C15—H15B	0.9800	C6—H6A	0.9900
C15—H15C	0.9800	C6—H6B	0.9900
C16—C17	1.532 (2)	C7—C8	1.538 (2)
C16—C8	1.542 (2)	C7—H7A	0.9900
C16—H16A	0.9900	C7—H7B	0.9900
C16—H16B	0.9900	C10—H10A	0.9900
C17—C18	1.503 (2)	C10—H10B	0.9900
			
C2—O1—C22	117.71 (12)	C19—C20—H20C	109.5
C2—C1—C9	107.24 (12)	H20A—C20—H20C	109.5
C2—C1—C8	113.22 (12)	H20B—C20—H20C	109.5
C9—C1—C8	109.15 (12)	C19—C21—H21A	109.5
C2—C1—H1	109.1	C19—C21—H21B	109.5
C9—C1—H1	109.1	H21A—C21—H21B	109.5
C8—C1—H1	109.1	C19—C21—H21C	109.5
O1—C2—C3	125.94 (14)	H21A—C21—H21C	109.5
O1—C2—C1	111.29 (12)	H21B—C21—H21C	109.5
C3—C2—C1	122.77 (13)	O1—C22—H22A	109.5
C12—C11—C10	127.01 (15)	O1—C22—H22B	109.5
C12—C11—H11	116.5	H22A—C22—H22B	109.5
C10—C11—H11	116.5	O1—C22—H22C	109.5
C11—C12—C13	120.73 (15)	H22A—C22—H22C	109.5
C11—C12—C14	125.18 (15)	H22B—C22—H22C	109.5
C13—C12—C14	114.07 (14)	C2—C3—C4	121.28 (14)
C12—C13—H13A	109.5	C2—C3—H3	119.4
C12—C13—H13B	109.5	C4—C3—H3	119.4
H13A—C13—H13B	109.5	O2—C4—C3	121.74 (14)
C12—C13—H13C	109.5	O2—C4—C5	119.27 (14)
H13A—C13—H13C	109.5	C3—C4—C5	118.99 (13)
H13B—C13—H13C	109.5	C9—C5—C4	109.70 (12)
C12—C14—H14A	109.5	C9—C5—C10	113.49 (12)
C12—C14—H14B	109.5	C4—C5—C10	111.23 (12)
H14A—C14—H14B	109.5	C9—C5—C6	105.56 (12)
C12—C14—H14C	109.5	C4—C5—C6	107.55 (12)
H14A—C14—H14C	109.5	C10—C5—C6	108.98 (12)
H14B—C14—H14C	109.5	C7—C6—C5	113.00 (12)
C8—C15—H15A	109.5	C7—C6—H6A	109.0
C8—C15—H15B	109.5	C5—C6—H6A	109.0
H15A—C15—H15B	109.5	C7—C6—H6B	109.0
C8—C15—H15C	109.5	C5—C6—H6B	109.0
H15A—C15—H15C	109.5	H6A—C6—H6B	107.8
H15B—C15—H15C	109.5	C6—C7—C8	114.29 (13)
C17—C16—C8	117.03 (13)	C6—C7—H7A	108.7
C17—C16—H16A	108.0	C8—C7—H7A	108.7
C8—C16—H16A	108.0	C6—C7—H7B	108.7
C17—C16—H16B	108.0	C8—C7—H7B	108.7
C8—C16—H16B	108.0	H7A—C7—H7B	107.6
H16A—C16—H16B	107.3	C15—C8—C7	109.82 (13)
C18—C17—C16	110.21 (14)	C15—C8—C16	110.96 (12)
C18—C17—H17A	109.6	C7—C8—C16	107.18 (12)
C16—C17—H17A	109.6	C15—C8—C1	107.69 (13)
C18—C17—H17B	109.6	C7—C8—C1	109.14 (12)
C16—C17—H17B	109.6	C16—C8—C1	112.04 (12)
H17A—C17—H17B	108.1	O3—C9—C1	122.82 (14)
C19—C18—C17	128.37 (16)	O3—C9—C5	124.51 (14)
C19—C18—H18	115.8	C1—C9—C5	112.66 (12)
C17—C18—H18	115.8	C11—C10—C5	116.15 (13)
C18—C19—C20	124.57 (16)	C11—C10—H10A	108.2
C18—C19—C21	121.04 (17)	C5—C10—H10A	108.2
C20—C19—C21	114.36 (16)	C11—C10—H10B	108.2
C19—C20—H20A	109.5	C5—C10—H10B	108.2
C19—C20—H20B	109.5	H10A—C10—H10B	107.4
H20A—C20—H20B	109.5		
			
C22—O1—C2—C3	0.3 (2)	C6—C7—C8—C15	67.77 (17)
C22—O1—C2—C1	−179.43 (13)	C6—C7—C8—C16	−171.61 (13)
C9—C1—C2—O1	147.89 (12)	C6—C7—C8—C1	−50.07 (17)
C8—C1—C2—O1	−91.66 (15)	C17—C16—C8—C15	−57.88 (18)
C9—C1—C2—C3	−31.9 (2)	C17—C16—C8—C7	−177.78 (13)
C8—C1—C2—C3	88.60 (17)	C17—C16—C8—C1	62.53 (17)
C10—C11—C12—C13	174.40 (15)	C2—C1—C8—C15	175.62 (12)
C10—C11—C12—C14	−4.1 (3)	C9—C1—C8—C15	−65.02 (15)
C8—C16—C17—C18	171.90 (13)	C2—C1—C8—C7	−65.22 (16)
C16—C17—C18—C19	107.5 (2)	C9—C1—C8—C7	54.14 (16)
C17—C18—C19—C20	1.7 (3)	C2—C1—C8—C16	53.33 (16)
C17—C18—C19—C21	−176.41 (16)	C9—C1—C8—C16	172.69 (12)
O1—C2—C3—C4	179.48 (14)	C2—C1—C9—O3	−122.71 (16)
C1—C2—C3—C4	−0.8 (2)	C8—C1—C9—O3	114.28 (16)
C2—C3—C4—O2	−170.94 (15)	C2—C1—C9—C5	58.69 (16)
C2—C3—C4—C5	8.2 (2)	C8—C1—C9—C5	−64.32 (15)
O2—C4—C5—C9	−162.44 (13)	C4—C5—C9—O3	128.82 (16)
C3—C4—C5—C9	18.39 (18)	C10—C5—C9—O3	3.7 (2)
O2—C4—C5—C10	−36.05 (19)	C6—C5—C9—O3	−115.57 (16)
C3—C4—C5—C10	144.78 (14)	C4—C5—C9—C1	−52.61 (16)
O2—C4—C5—C6	83.22 (16)	C10—C5—C9—C1	−177.70 (12)
C3—C4—C5—C6	−95.96 (15)	C6—C5—C9—C1	63.01 (15)
C9—C5—C6—C7	−55.72 (16)	C12—C11—C10—C5	117.26 (17)
C4—C5—C6—C7	61.36 (16)	C9—C5—C10—C11	68.76 (17)
C10—C5—C6—C7	−177.95 (13)	C4—C5—C10—C11	−55.51 (18)
C5—C6—C7—C8	52.82 (18)	C6—C5—C10—C11	−173.92 (13)

### 4-tert-Butyl-4-hydroxy-2-methoxy-8-methyl-7-(3-methylbut-2-en-1-yl)-8-(4-methylpent-3-en-1-yl)bicyclo[3.3.1]non-2-en-9-one (4) Crystal data

**Table:**

C_26_H_42_O_3_	*F*(000) = 888
*M**_r_* = 402.59	*D*_x_ = 1.080 Mg m^−^^3^
Monoclinic, *P*2_1_/*c*	Mo *K*α radiation, λ = 0.71073 Å
*a* = 15.1165 (5) Å	Cell parameters from 9769 reflections
*b* = 13.9068 (4) Å	θ = 2.9–27.1°
*c* = 12.8150 (4) Å	µ = 0.07 mm^−^^1^
β = 113.218 (1)°	*T* = 143 K
*V* = 2475.81 (13) Å^3^	Prism, colourless
*Z* = 4	0.26 × 0.16 × 0.08 mm

### 4-tert-Butyl-4-hydroxy-2-methoxy-8-methyl-7-(3-methylbut-2-en-1-yl)-8-(4-methylpent-3-en-1-yl)bicyclo[3.3.1]non-2-en-9-one (4) Data collection

**Table:**

Bruker D8 VENTURE PHOTON II diffractometer	4697 reflections with *I* > 2σ(*I*)
Radiation source: INCOATEC IÂµS microfocus sealed tube	*R*_int_ = 0.048
φ and ω scans	θ_max_ = 27.1°, θ_min_ = 2.1°
Absorption correction: multi-scan (SADABS; Krause *et al.*, 2015)	*h* = −19→19
*T*_min_ = 0.704, *T*_max_ = 0.746	*k* = −16→17
87917 measured reflections	*l* = −16→16
5484 independent reflections	

### 4-tert-Butyl-4-hydroxy-2-methoxy-8-methyl-7-(3-methylbut-2-en-1-yl)-8-(4-methylpent-3-en-1-yl)bicyclo[3.3.1]non-2-en-9-one (4) Refinement

**Table:**

Refinement on *F*^2^	Primary atom site location: structure-invariant direct methods
Least-squares matrix: full	Secondary atom site location: difference Fourier map
*R*[*F*^2^ > 2σ(*F*^2^)] = 0.040	Hydrogen site location: mixed
*wR*(*F*^2^) = 0.108	H atoms treated by a mixture of independent and constrained refinement
*S* = 1.05	*w* = 1/[σ^2^(*F*_o_^2^) + (0.049*P*)^2^ + 0.8245*P*] where *P* = (*F*_o_^2^ + 2*F*_c_^2^)/3
5484 reflections	(Δ/σ)_max_ < 0.001
274 parameters	Δρ_max_ = 0.29 e Å^−^^3^
1 restraint	Δρ_min_ = −0.20 e Å^−^^3^

### 4-tert-Butyl-4-hydroxy-2-methoxy-8-methyl-7-(3-methylbut-2-en-1-yl)-8-(4-methylpent-3-en-1-yl)bicyclo[3.3.1]non-2-en-9-one (4) Special details

**Table:**

Geometry. All esds (except the esd in the dihedral angle between two l.s. planes) are estimated using the full covariance matrix. The cell esds are taken into account individually in the estimation of esds in distances, angles and torsion angles; correlations between esds in cell parameters are only used when they are defined by crystal symmetry. An approximate (isotropic) treatment of cell esds is used for estimating esds involving l.s. planes.

### 4-tert-Butyl-4-hydroxy-2-methoxy-8-methyl-7-(3-methylbut-2-en-1-yl)-8-(4-methylpent-3-en-1-yl)bicyclo[3.3.1]non-2-en-9-one (4) Fractional atomic coordinates and isotropic or equivalent isotropic displacement parameters (Å^2^)

**Table:**

	*x*	*y*	*z*	*U*_iso_*/*U*_eq_	
O1	0.58921 (6)	0.47108 (5)	0.43047 (6)	0.02376 (18)	
O2	0.51178 (6)	0.14696 (6)	0.37950 (7)	0.02551 (18)	
H2	0.5478 (10)	0.1689 (11)	0.3487 (12)	0.038*	
O3	0.61221 (6)	0.30868 (6)	0.73617 (6)	0.02674 (19)	
C1	0.65561 (7)	0.36534 (7)	0.58638 (9)	0.0191 (2)	
H1	0.668130	0.427362	0.629287	0.023*	
C2	0.58011 (7)	0.38158 (7)	0.46865 (9)	0.0187 (2)	
C3	0.51410 (8)	0.31676 (8)	0.41130 (9)	0.0202 (2)	
H3	0.470212	0.333360	0.336969	0.024*	
C4	0.50402 (8)	0.21886 (7)	0.45585 (9)	0.0198 (2)	
C5	0.58310 (8)	0.20205 (7)	0.57770 (9)	0.0202 (2)	
H5	0.556494	0.158196	0.620023	0.024*	
C6	0.67741 (8)	0.15818 (8)	0.57903 (10)	0.0229 (2)	
H6A	0.718889	0.140699	0.658210	0.028*	
H6B	0.661579	0.098106	0.533934	0.028*	
C7	0.73503 (8)	0.22364 (8)	0.53245 (9)	0.0210 (2)	
H7	0.695333	0.232637	0.449626	0.025*	
C8	0.75364 (7)	0.32539 (8)	0.58735 (9)	0.0205 (2)	
C9	0.61403 (7)	0.29414 (8)	0.64324 (9)	0.0197 (2)	
C10	0.82710 (8)	0.16941 (9)	0.54227 (10)	0.0276 (2)	
H10A	0.869428	0.213478	0.522244	0.033*	
H10B	0.862400	0.148432	0.621810	0.033*	
C11	0.80410 (8)	0.08302 (9)	0.46537 (10)	0.0274 (2)	
H11	0.752582	0.090867	0.393411	0.033*	
C12	0.84620 (9)	−0.00269 (9)	0.48468 (11)	0.0318 (3)	
C13	0.92703 (13)	−0.03188 (12)	0.59291 (14)	0.0542 (4)	
H13A	0.982739	−0.051060	0.576768	0.081*	
H13B	0.906467	−0.086094	0.626814	0.081*	
H13C	0.944762	0.022430	0.645939	0.081*	
C14	0.81468 (12)	−0.07908 (11)	0.39421 (15)	0.0473 (4)	
H14A	0.758079	−0.056563	0.329342	0.071*	
H14B	0.798374	−0.137879	0.424891	0.071*	
H14C	0.867064	−0.092552	0.369481	0.071*	
C15	0.82707 (8)	0.32225 (9)	0.71095 (10)	0.0272 (2)	
H15A	0.890430	0.304716	0.712693	0.041*	
H15B	0.806850	0.274391	0.753214	0.041*	
H15C	0.830812	0.385648	0.745833	0.041*	
C16	0.78978 (8)	0.39199 (8)	0.51636 (10)	0.0255 (2)	
H16A	0.848819	0.363159	0.513943	0.031*	
H16B	0.740565	0.392664	0.437600	0.031*	
C17	0.81238 (9)	0.49662 (9)	0.55609 (11)	0.0331 (3)	
H17A	0.756181	0.525580	0.565571	0.040*	
H17B	0.867730	0.498447	0.630446	0.040*	
C18	0.83575 (9)	0.55326 (9)	0.47056 (12)	0.0347 (3)	
H18	0.790871	0.547761	0.394072	0.042*	
C19	0.91070 (9)	0.60995 (9)	0.48753 (12)	0.0336 (3)	
C20	0.92557 (11)	0.65611 (12)	0.38952 (14)	0.0478 (4)	
H20A	0.870648	0.641423	0.318709	0.072*	
H20B	0.984685	0.631054	0.385359	0.072*	
H20C	0.931060	0.725920	0.400634	0.072*	
C21	0.98769 (13)	0.63083 (14)	0.60163 (15)	0.0597 (5)	
H21A	1.048455	0.602150	0.606663	0.090*	
H21B	0.969451	0.603470	0.660797	0.090*	
H21C	0.995622	0.700564	0.612238	0.090*	
C22	0.53199 (10)	0.49151 (9)	0.31424 (10)	0.0335 (3)	
H22A	0.549538	0.554761	0.294582	0.050*	
H22B	0.463789	0.491517	0.302071	0.050*	
H22C	0.543299	0.442285	0.266167	0.050*	
C23	0.40054 (8)	0.20444 (8)	0.45525 (10)	0.0244 (2)	
C24	0.38671 (9)	0.26723 (9)	0.54585 (11)	0.0298 (3)	
H24A	0.402128	0.334181	0.535978	0.045*	
H24B	0.429455	0.244724	0.621440	0.045*	
H24C	0.319688	0.262973	0.538096	0.045*	
C25	0.32344 (9)	0.23243 (11)	0.33952 (11)	0.0378 (3)	
H25A	0.335664	0.198822	0.279316	0.057*	
H25B	0.325506	0.302032	0.328713	0.057*	
H25C	0.259812	0.214354	0.336505	0.057*	
C26	0.38494 (9)	0.09832 (9)	0.47587 (12)	0.0345 (3)	
H26A	0.320208	0.089861	0.474739	0.052*	
H26B	0.432888	0.078417	0.549936	0.052*	
H26C	0.391761	0.058869	0.416106	0.052*	

### 4-tert-Butyl-4-hydroxy-2-methoxy-8-methyl-7-(3-methylbut-2-en-1-yl)-8-(4-methylpent-3-en-1-yl)bicyclo[3.3.1]non-2-en-9-one (4) Atomic displacement parameters (Å^2^)

**Table:**

	*U*^11^	*U*^22^	*U*^33^	*U*^12^	*U*^13^	*U*^23^
O1	0.0315 (4)	0.0169 (4)	0.0221 (4)	−0.0007 (3)	0.0097 (3)	0.0035 (3)
O2	0.0346 (4)	0.0228 (4)	0.0247 (4)	−0.0073 (3)	0.0177 (3)	−0.0078 (3)
O3	0.0294 (4)	0.0363 (5)	0.0168 (4)	−0.0006 (3)	0.0115 (3)	−0.0018 (3)
C1	0.0230 (5)	0.0177 (5)	0.0171 (5)	−0.0021 (4)	0.0086 (4)	−0.0023 (4)
C2	0.0236 (5)	0.0171 (5)	0.0183 (5)	0.0011 (4)	0.0112 (4)	0.0010 (4)
C3	0.0239 (5)	0.0213 (5)	0.0155 (5)	−0.0003 (4)	0.0079 (4)	0.0008 (4)
C4	0.0247 (5)	0.0186 (5)	0.0181 (5)	−0.0039 (4)	0.0107 (4)	−0.0032 (4)
C5	0.0246 (5)	0.0191 (5)	0.0196 (5)	−0.0018 (4)	0.0117 (4)	0.0021 (4)
C6	0.0268 (5)	0.0188 (5)	0.0250 (5)	0.0012 (4)	0.0121 (4)	0.0027 (4)
C7	0.0218 (5)	0.0210 (5)	0.0213 (5)	0.0004 (4)	0.0096 (4)	−0.0002 (4)
C8	0.0204 (5)	0.0219 (5)	0.0196 (5)	−0.0016 (4)	0.0084 (4)	−0.0009 (4)
C9	0.0193 (5)	0.0237 (5)	0.0160 (5)	0.0018 (4)	0.0068 (4)	0.0013 (4)
C10	0.0239 (5)	0.0282 (6)	0.0318 (6)	0.0026 (4)	0.0120 (5)	−0.0021 (5)
C11	0.0279 (6)	0.0303 (6)	0.0269 (6)	0.0033 (5)	0.0140 (5)	−0.0008 (5)
C12	0.0348 (6)	0.0296 (6)	0.0382 (7)	0.0040 (5)	0.0222 (6)	0.0022 (5)
C13	0.0608 (10)	0.0476 (9)	0.0506 (9)	0.0248 (8)	0.0181 (8)	0.0114 (7)
C14	0.0577 (9)	0.0333 (7)	0.0629 (10)	0.0009 (6)	0.0364 (8)	−0.0108 (7)
C15	0.0239 (5)	0.0318 (6)	0.0230 (5)	−0.0012 (4)	0.0062 (4)	−0.0010 (5)
C16	0.0237 (5)	0.0267 (6)	0.0287 (6)	−0.0035 (4)	0.0131 (5)	0.0012 (4)
C17	0.0332 (6)	0.0293 (6)	0.0369 (7)	−0.0111 (5)	0.0140 (5)	−0.0011 (5)
C18	0.0268 (6)	0.0327 (6)	0.0399 (7)	−0.0055 (5)	0.0082 (5)	0.0079 (5)
C19	0.0278 (6)	0.0265 (6)	0.0461 (8)	−0.0023 (5)	0.0141 (5)	0.0053 (5)
C20	0.0330 (7)	0.0471 (8)	0.0615 (10)	−0.0042 (6)	0.0167 (7)	0.0222 (7)
C21	0.0559 (10)	0.0690 (12)	0.0518 (10)	−0.0365 (9)	0.0186 (8)	−0.0119 (8)
C22	0.0497 (8)	0.0228 (6)	0.0247 (6)	0.0035 (5)	0.0109 (5)	0.0072 (5)
C23	0.0237 (5)	0.0264 (6)	0.0250 (5)	−0.0062 (4)	0.0115 (4)	−0.0040 (4)
C24	0.0262 (6)	0.0340 (6)	0.0332 (6)	−0.0033 (5)	0.0160 (5)	−0.0062 (5)
C25	0.0253 (6)	0.0515 (8)	0.0318 (7)	−0.0085 (5)	0.0062 (5)	−0.0019 (6)
C26	0.0366 (7)	0.0293 (6)	0.0444 (7)	−0.0127 (5)	0.0232 (6)	−0.0050 (5)

### 4-tert-Butyl-4-hydroxy-2-methoxy-8-methyl-7-(3-methylbut-2-en-1-yl)-8-(4-methylpent-3-en-1-yl)bicyclo[3.3.1]non-2-en-9-one (4) Geometric parameters (Å, º)

**Table:**

O1—C2	1.3640 (12)	C14—H14B	0.9800
O1—C22	1.4254 (14)	C14—H14C	0.9800
O2—C4	1.4357 (12)	C15—H15A	0.9800
O2—H2	0.846 (9)	C15—H15B	0.9800
O3—C9	1.2189 (13)	C15—H15C	0.9800
C1—C9	1.5058 (14)	C16—C17	1.5350 (17)
C1—C2	1.5073 (14)	C16—H16A	0.9900
C1—C8	1.5781 (14)	C16—H16B	0.9900
C1—H1	1.0000	C17—C18	1.5005 (18)
C2—C3	1.3305 (15)	C17—H17A	0.9900
C3—C4	1.5071 (14)	C17—H17B	0.9900
C3—H3	0.9500	C18—C19	1.3261 (17)
C4—C5	1.5652 (14)	C18—H18	0.9500
C4—C23	1.5741 (15)	C19—C21	1.495 (2)
C5—C9	1.5016 (15)	C19—C20	1.5042 (19)
C5—C6	1.5446 (15)	C20—H20A	0.9800
C5—H5	1.0000	C20—H20B	0.9800
C6—C7	1.5342 (15)	C20—H20C	0.9800
C6—H6A	0.9900	C21—H21A	0.9800
C6—H6B	0.9900	C21—H21B	0.9800
C7—C10	1.5439 (15)	C21—H21C	0.9800
C7—C8	1.5558 (15)	C22—H22A	0.9800
C7—H7	1.0000	C22—H22B	0.9800
C8—C15	1.5350 (15)	C22—H22C	0.9800
C8—C16	1.5414 (15)	C23—C24	1.5315 (16)
C10—C11	1.5051 (16)	C23—C25	1.5319 (17)
C10—H10A	0.9900	C23—C26	1.5338 (16)
C10—H10B	0.9900	C24—H24A	0.9800
C11—C12	1.3277 (17)	C24—H24B	0.9800
C11—H11	0.9500	C24—H24C	0.9800
C12—C13	1.498 (2)	C25—H25A	0.9800
C12—C14	1.5047 (19)	C25—H25B	0.9800
C13—H13A	0.9800	C25—H25C	0.9800
C13—H13B	0.9800	C26—H26A	0.9800
C13—H13C	0.9800	C26—H26B	0.9800
C14—H14A	0.9800	C26—H26C	0.9800
			
C2—O1—C22	116.82 (9)	H14A—C14—H14C	109.5
C4—O2—H2	107.9 (11)	H14B—C14—H14C	109.5
C9—C1—C2	106.68 (8)	C8—C15—H15A	109.5
C9—C1—C8	109.54 (8)	C8—C15—H15B	109.5
C2—C1—C8	113.50 (8)	H15A—C15—H15B	109.5
C9—C1—H1	109.0	C8—C15—H15C	109.5
C2—C1—H1	109.0	H15A—C15—H15C	109.5
C8—C1—H1	109.0	H15B—C15—H15C	109.5
C3—C2—O1	125.44 (10)	C17—C16—C8	117.27 (10)
C3—C2—C1	123.95 (9)	C17—C16—H16A	108.0
O1—C2—C1	110.61 (9)	C8—C16—H16A	108.0
C2—C3—C4	124.65 (9)	C17—C16—H16B	108.0
C2—C3—H3	117.7	C8—C16—H16B	108.0
C4—C3—H3	117.7	H16A—C16—H16B	107.2
O2—C4—C3	108.86 (8)	C18—C17—C16	109.94 (11)
O2—C4—C5	109.90 (8)	C18—C17—H17A	109.7
C3—C4—C5	111.18 (8)	C16—C17—H17A	109.7
O2—C4—C23	104.83 (8)	C18—C17—H17B	109.7
C3—C4—C23	111.22 (9)	C16—C17—H17B	109.7
C5—C4—C23	110.64 (8)	H17A—C17—H17B	108.2
C9—C5—C6	104.37 (8)	C19—C18—C17	128.62 (13)
C9—C5—C4	112.18 (8)	C19—C18—H18	115.7
C6—C5—C4	114.12 (9)	C17—C18—H18	115.7
C9—C5—H5	108.7	C18—C19—C21	124.14 (13)
C6—C5—H5	108.7	C18—C19—C20	121.07 (13)
C4—C5—H5	108.7	C21—C19—C20	114.76 (12)
C7—C6—C5	115.11 (9)	C19—C20—H20A	109.5
C7—C6—H6A	108.5	C19—C20—H20B	109.5
C5—C6—H6A	108.5	H20A—C20—H20B	109.5
C7—C6—H6B	108.5	C19—C20—H20C	109.5
C5—C6—H6B	108.5	H20A—C20—H20C	109.5
H6A—C6—H6B	107.5	H20B—C20—H20C	109.5
C6—C7—C10	108.08 (9)	C19—C21—H21A	109.5
C6—C7—C8	113.13 (9)	C19—C21—H21B	109.5
C10—C7—C8	114.26 (9)	H21A—C21—H21B	109.5
C6—C7—H7	107.0	C19—C21—H21C	109.5
C10—C7—H7	107.0	H21A—C21—H21C	109.5
C8—C7—H7	107.0	H21B—C21—H21C	109.5
C15—C8—C16	110.22 (9)	O1—C22—H22A	109.5
C15—C8—C7	111.66 (9)	O1—C22—H22B	109.5
C16—C8—C7	108.81 (9)	H22A—C22—H22B	109.5
C15—C8—C1	108.24 (8)	O1—C22—H22C	109.5
C16—C8—C1	109.51 (9)	H22A—C22—H22C	109.5
C7—C8—C1	108.35 (8)	H22B—C22—H22C	109.5
O3—C9—C5	124.47 (10)	C24—C23—C25	108.05 (10)
O3—C9—C1	122.56 (10)	C24—C23—C26	109.99 (10)
C5—C9—C1	112.80 (8)	C25—C23—C26	107.54 (10)
C11—C10—C7	111.61 (9)	C24—C23—C4	110.86 (9)
C11—C10—H10A	109.3	C25—C23—C4	110.35 (9)
C7—C10—H10A	109.3	C26—C23—C4	109.98 (9)
C11—C10—H10B	109.3	C23—C24—H24A	109.5
C7—C10—H10B	109.3	C23—C24—H24B	109.5
H10A—C10—H10B	108.0	H24A—C24—H24B	109.5
C12—C11—C10	128.65 (12)	C23—C24—H24C	109.5
C12—C11—H11	115.7	H24A—C24—H24C	109.5
C10—C11—H11	115.7	H24B—C24—H24C	109.5
C11—C12—C13	124.87 (13)	C23—C25—H25A	109.5
C11—C12—C14	120.48 (13)	C23—C25—H25B	109.5
C13—C12—C14	114.64 (12)	H25A—C25—H25B	109.5
C12—C13—H13A	109.5	C23—C25—H25C	109.5
C12—C13—H13B	109.5	H25A—C25—H25C	109.5
H13A—C13—H13B	109.5	H25B—C25—H25C	109.5
C12—C13—H13C	109.5	C23—C26—H26A	109.5
H13A—C13—H13C	109.5	C23—C26—H26B	109.5
H13B—C13—H13C	109.5	H26A—C26—H26B	109.5
C12—C14—H14A	109.5	C23—C26—H26C	109.5
C12—C14—H14B	109.5	H26A—C26—H26C	109.5
H14A—C14—H14B	109.5	H26B—C26—H26C	109.5
C12—C14—H14C	109.5		
			
C22—O1—C2—C3	−9.12 (16)	C9—C1—C8—C7	54.45 (10)
C22—O1—C2—C1	171.02 (9)	C2—C1—C8—C7	−64.65 (11)
C9—C1—C2—C3	−27.68 (14)	C6—C5—C9—O3	−111.33 (11)
C8—C1—C2—C3	93.05 (12)	C4—C5—C9—O3	124.61 (11)
C9—C1—C2—O1	152.17 (8)	C6—C5—C9—C1	64.07 (11)
C8—C1—C2—O1	−87.10 (10)	C4—C5—C9—C1	−59.99 (11)
O1—C2—C3—C4	−178.78 (9)	C2—C1—C9—O3	−128.15 (10)
C1—C2—C3—C4	1.06 (17)	C8—C1—C9—O3	108.61 (11)
C2—C3—C4—O2	−122.62 (11)	C2—C1—C9—C5	56.35 (11)
C2—C3—C4—C5	−1.41 (14)	C8—C1—C9—C5	−66.88 (11)
C2—C3—C4—C23	122.37 (11)	C6—C7—C10—C11	66.44 (12)
O2—C4—C5—C9	150.21 (8)	C8—C7—C10—C11	−166.66 (9)
C3—C4—C5—C9	29.61 (12)	C7—C10—C11—C12	−142.25 (12)
C23—C4—C5—C9	−94.50 (10)	C10—C11—C12—C13	1.6 (2)
O2—C4—C5—C6	31.76 (11)	C10—C11—C12—C14	−177.64 (12)
C3—C4—C5—C6	−88.83 (10)	C15—C8—C16—C17	−57.38 (13)
C23—C4—C5—C6	147.06 (9)	C7—C8—C16—C17	179.86 (10)
C9—C5—C6—C7	−55.79 (11)	C1—C8—C16—C17	61.59 (12)
C4—C5—C6—C7	67.01 (12)	C8—C16—C17—C18	−173.70 (10)
C5—C6—C7—C10	178.96 (9)	C16—C17—C18—C19	−129.72 (15)
C5—C6—C7—C8	51.40 (12)	C17—C18—C19—C21	−2.1 (2)
C6—C7—C8—C15	71.38 (11)	C17—C18—C19—C20	175.86 (14)
C10—C7—C8—C15	−52.86 (12)	O2—C4—C23—C24	172.12 (9)
C6—C7—C8—C16	−166.73 (9)	C3—C4—C23—C24	−70.39 (12)
C10—C7—C8—C16	69.03 (11)	C5—C4—C23—C24	53.69 (12)
C6—C7—C8—C1	−47.73 (11)	O2—C4—C23—C25	−68.21 (11)
C10—C7—C8—C1	−171.97 (9)	C3—C4—C23—C25	49.28 (12)
C9—C1—C8—C15	−66.80 (11)	C5—C4—C23—C25	173.36 (9)
C2—C1—C8—C15	174.10 (9)	O2—C4—C23—C26	50.27 (11)
C9—C1—C8—C16	173.01 (8)	C3—C4—C23—C26	167.75 (9)
C2—C1—C8—C16	53.90 (11)	C5—C4—C23—C26	−68.16 (11)

### 4-tert-Butyl-4-hydroxy-2-methoxy-8-methyl-7-(3-methylbut-2-en-1-yl)-8-(4-methylpent-3-en-1-yl)bicyclo[3.3.1]non-2-en-9-one (4) Hydrogen-bond geometry (Å, º)

**Table:**

*D*—H···*A*	*D*—H	H···*A*	*D*···*A*	*D*—H···*A*
O2—H2···O3^i^	0.85 (1)	2.06 (1)	2.8736 (11)	162 (2)

Symmetry code: (i) *x*, −*y*+1/2, *z*−1/2.
